# The impact of specialist resource centres on autistic pupils’ experience of mainstream school

**DOI:** 10.1177/13623613261426099

**Published:** 2026-03-13

**Authors:** Anna Cook, Alice Boddy

**Affiliations:** 1 University of Surrey, UK

**Keywords:** autism, inclusive education, peer support, psychological well-being, resource base provision, school attendance, school belonging, social inclusion, specialist resource centre, teacher support

## Abstract

This study investigated how autistic pupils’ psychological, social and educational outcomes differed in contrasting mainstream provisions. Using a three-year longitudinal quasi-experimental design, outcomes were compared across three placements: placement in a specialist resource centre, mainstream placement in specialist resource centre host schools, and placement in mainstream schools without a specialist resource centre. Autistic pupils (*N* = 119, aged 11–14) and matched non-autistic peers (*N* = 119) completed surveys and schools provided attainment, attendance, and exclusion data. Placement type showed no main effects on psychosocial outcomes in mixed analyses of variance, indicating limited evidence for differential change by placement alone, but hierarchical regressions indicated that specialist resource centre placement was statistically associated with higher academic attainment and stronger school belonging than mainstream schools without a specialist resource centre, and greater perceived teacher support than mainstream placement in specialist resource centre host schools. Attendance disparities for specialist resource centre pupils were smaller than national averages for autistic pupils, though attendance remained below whole-school averages. Perceived peer and teacher support were most strongly associated with positive outcomes across models, highlighting relational inclusion as a potential mechanism. Findings underscore the value of positive relationships in fostering belonging, reducing disparities, and promoting well-being for autistic pupils. Overall, specialist resource centre approaches may be associated with academic and belonging advantages, while relational inclusion appears central to positive outcomes.

**Lay abstract:**

This study explored how support through specialist resource centres can help autistic pupils in mainstream secondary schools. Specialist resource centres are supportive spaces within regular schools that offer extra help, trained staff who understand autism, and a calm environment, while also keeping pupils included in the wider school community. Although national policies aim to make schools more inclusive, many autistic pupils still face challenges such as anxiety, social challenges and school absence. This study followed 119 autistic pupils aged 11–14 across seven schools for three years. It compared three groups: pupils in specialist resource centres, autistic pupils in the same schools but not placed in the Centres and autistic pupils in mainstream schools without a Centre. Comparisons were also made with non-autistic pupils from the same schools. The study found that specialist resource centre placement was linked to better academic progress for pupils in this sample and a stronger sense of belonging than other placements. Even so, placement on its own did not lead to clear differences in most areas of well-being, and some results were based on smaller samples, so they should be treated carefully. Specialist resource centre pupils also felt more supported by teachers, and they reported higher levels of happiness and fewer peer problems than non-autistic peers. However, because this information was gathered at one point in time, the findings do not prove that placement caused these differences, though they leave open the possibility that placement may have had an impact. Pupils in specialist resource centre placements appeared to have better attendance than autistic pupils nationally, although attendance was still not as high as whole-school averages. The most important factor linked to positive outcomes was feeling supported by teachers and classmates. These results suggest specialist resource centres may offer the greatest benefits within mainstream schools, when they help pupils build supportive peer and teacher relationships.

## Introduction

In recent years, there has been growing recognition of the need to better understand the educational experiences and well-being of autistic pupils in mainstream educational settings. As a result of worldwide educational inclusion policies (United Nations Educational, Scientific & Cultural Organization (UNESCO), 2009) and earlier global commitments such as the Salamanca Statement (UNESCO, 1994), many more autistic children are now educated in mainstream schools, particularly in Europe and America. Yet international evidence continues to show persistent gaps between inclusive policy and practice, and highly variable quality of support for disabled and autistic students across high-income contexts (Ainscow, 2020; Falkmer et al., 2015; Keen et al., 2016; Pfeffer, 2015; Tobias, 2009). In the United Kingdom, where this study took place, over 236,000 autistic pupils are currently educated in state-funded schools across England (Department for Education (DfE), 2024b), with approximately 73% attending mainstream schools (National Autistic Society (NAS), 2021). Despite inclusive intent, autistic pupils continue to face barriers that affect academic progress, social engagement and mental health (Billington et al., 2024; Keen et al., 2016; Lai et al., 2019; Maiano et al., 2016).

The landscape of SEND policy in the United Kingdom has also undergone significant change. The 2014 SEND Code of Practice (DfE & Department of Health and Social Care (DHSC), 2014) sought to strengthen inclusion and improve support for pupils with additional needs in mainstream schools, building on earlier reforms such as the Education Act 1981 and subsequent critiques of inclusion policy (Education Act, 1981; Warnock, 2005). More recent reforms aim to improve consistency and accountability (DfE, 2022, 2023). However, implementation has been slower than expected, and schools report ongoing challenges in meeting autistic pupils’ needs, including insufficient resources, limited staff training, and the complexity of supporting diverse profiles of need (Bond et al., 2016; Hamilton & Cook, 2026; Horgan et al., 2022). These systemic weaknesses sit behind continuing debates about whether specialist placements inevitably undermine inclusion, or whether flexible hybrid arrangements can offer a more responsive form of mainstream education (Goodall, 2018b; Hebron & Bond, 2017). Understanding placement effects therefore requires attention not only to setting type, but also to the specific psychological, social and educational challenges faced by autistic pupils in mainstream schools.

Across the literature, autistic pupils consistently report elevated mental health vulnerability in mainstream settings. Relative to non-autistic peers, they show higher rates of internalising symptoms such as anxiety and depression, and difficulties with emotion regulation (Ashburner et al., 2010; Avenevoli et al., 2015; Greenlee et al., 2016; Hebron & Humphrey, 2014; Keith et al., 2019; McGinnity et al., 2005; Merikangas et al., 2010; Mertens et al., 2017; Ridgway et al., 2024; Van Steensel et al., 2011). These difficulties are linked to sensory overload, social demands, academic pressure, rigid expectations and limited autism awareness in school cultures (Cook et al., 2018; Goodall, 2018a, 2018b; Humphrey & Symes, 2010). Autistic-led and lived-experience research further emphasises that well-being is tightly linked to authenticity, acceptance and relief from masking pressures at school (Botha et al., 2022; L. Chapman et al., 2022; den Houting et al., 2021; Tierney et al., 2016). Identifying what supports flourishing in mainstream contexts is therefore a key inclusion priority (Keyes, 2007; Lippman et al., 2014; Seligman, 2011).

Social inclusion is another major challenge. Autistic pupils often experience difficulties forming and maintaining friendships (Cook et al., 2016; Goodall, 2018a; Goodall & MacKenzie, 2019), alongside heightened loneliness and social anxiety (Goodall, 2018b). At the same time, positive peer relationships can substantially enhance belonging and school engagement (Aubineau & Blicharska, 2020; Richter et al., 2020). Supportive teacher–pupil and peer relationships are repeatedly linked to stronger school connectedness, motivation and mental health, indicating that relational inclusion is a central mechanism through which broader well-being outcomes may be shaped (Carter, 2021; Cresswell et al., 2019; Jennings & Greenberg, 2009; Newcomb & Bagwell, 1995; Roorda et al., 2011; Shochet et al., 2006; Tierney et al., 2016; Wentzel et al., 2010). However, autistic pupils frequently report receiving limited targeted social support in mainstream classrooms (Humphrey & Symes, 2010), raising questions about how different provision models foster – or fail to foster – belonging.

Peer attitudes and school social norms also create barriers. Stigma, rejection and bullying are common experiences (Carrington et al., 2017; Cook et al., 2018; Goodall, 2018a, 2019; Hebron et al., 2015; Humphrey & Lewis, 2008; Poon et al., 2014). Autistic girls in particular may mask differences to avoid negative attention, a strategy that can carry mental health costs (Cook et al., 2018; Dean et al., 2017; Halsall et al., 2021; Hull et al., 2020; Tierney et al., 2016). Because many school cultures remain organised around non-autistic norms, autistic pupils often feel excluded from full participation (Botha et al., 2022; Carter, 2021). Belonging – feeling accepted and valued as one’s authentic self – is therefore a critical outcome for evaluating inclusive practice (Carter, 2021; Krieger et al., 2018), though it is unclear whether mainstream settings can consistently nurture belonging without substantial structural and cultural change (Falkmer et al., 2015; Goodall, 2018b; Horgan et al., 2022; Tobias, 2009).

Academic progress for autistic pupils can be impeded by fast-paced lessons, heavy workloads and rigid curricula that fail to accommodate individual needs (Aubineau & Blicharska, 2020; Goodall, 2018a; Neal & Frederickson, 2016; Poon et al., 2014; Saggers, 2015; Saggers et al., 2011). These challenges interact with wider indicators of school engagement, including attendance and exclusion. Autistic pupils are disproportionately affected by school distress and persistent absence (Connolly et al., 2023; DfE & Office for National Statistics (ONS), 2017) and face elevated exclusion risk, often linked to unmet needs or misinterpretation of neurodevelopmental behaviour (Aitken & Wang, 2019; Brede et al., 2017; E. Chapman, 2023; Ferguson, 2021; G. Lindsay et al., 2016). Attendance and exclusion therefore provide important equity markers for assessing provision impacts, while also pointing to systemic shortcomings that may not be solved by placement alone.

Physical and sensory aspects of mainstream schools further shape these trajectories. Crowded corridors, noise and unpredictable routines can be overwhelming and disruptive to learning and regulation (Goodall, 2018b, 2019; Horgan et al., 2022; Neal & Frederickson, 2016; Saggers, 2015). Autistic pupils commonly describe the need for quieter spaces and smaller-scale settings to manage anxiety and sensory load (Goodall, 2018a; Hill, 2014; McAllister & Sloan, 2016). Taken together, this literature suggests that placement effects may operate through the extent to which settings provide relational support, sensory adaptation and predictability, but the causal pathways and how they differ across provision types remain insufficiently understood (Carter, 2021; Jennings & Greenberg, 2009; Newcomb & Bagwell, 1995; Roorda et al., 2011; Shochet et al., 2006).

In this context, specialist support models have been developed to bridge the gap between inclusion policy and practice. Specialist resource centres (SRCs) aim to provide a calm, structured environment with tailored support for autistic pupils. In the United Kingdom, SRCs (also termed ‘specialist resource bases’ or ‘units’) are typically located within mainstream schools and offer small-group or individual teaching, access to autism-friendly spaces and support from staff with specialist expertise, while enabling pupils to attend mainstream classes for part of the school day (Moore, 2016; NAS, 2015). Comparable hybrid models exist internationally, including satellite or resource-class provision in Australia (Keane et al., 2012; Roberts et al., 2008), Japan (Ito et al., 2023) and Mexico (García-Cedillo et al., 2015). Decisions about SRC access also matter at scale: a national review for the DfE’s (2024) Delivering Better Value in SEND programme reported low parental confidence in mainstream provision and found that only 38% of autistic children were in their ideal educational setting. Although 14.7% were judged best suited to SRCs, only 2.9% were placed in them, and the proportion of SRC placements has fallen over time (DfE, 2024). Similar concerns about limited access to appropriate provision and long waits for suitable placements are reported across national contexts (Falkmer et al., 2015; G. Lindsay et al., 2016; Moore, 2016; NAS, 2015).

Existing evidence on SRCs is promising but limited. A small number of qualitative studies suggest that SRCs may offer protective features such as secure spaces, personalised support, curricular flexibility and opportunities to build confidence, progress, motivation and belonging (Bond & Hebron, 2016; Croydon et al., 2019; Hebron & Bond, 2017). Quantitative work also indicates potential benefits for peer attitudes (Cook et al., 2020). However, larger-scale, longitudinal evaluations with appropriate mainstream comparison groups remain scarce, leaving unresolved questions about the longer-term implications of SRC placements for inclusion, identity and belonging (Botha et al., 2022; Brede et al., 2017; Croydon et al., 2019). Moreover, previous studies have rarely measured psychosocial outcomes alongside educational indicators and perceived support within the same cohort, limiting understanding of whether and how relational mechanisms may explain any placement differences.

Building on the limited comparative evidence, this study forms part of a mixed-methods evaluation examining how different mainstream placement models relate to autistic pupils’ adjustment over time. (Complementary qualitative analyses from the same evaluation, drawing on pupils’, parents’ and staff perspectives across the same schools are reported elsewhere and help contextualise the quantitative findings (Boddy & Cook, 2026).) Using a three-year longitudinal cohort design, this study evaluates whether placement type – SRCs, mainstream placement within SRC host schools (M-SRC) and mainstream schools without SRCs (N-SRC) – is associated with distinctive trajectories in psychological well-being, social inclusion and belonging, educational progress, attendance, exclusions, and perceived teacher and peer support. By evaluating the combined contribution of placement and perceived support, the study responds to the United Kingdom and international calls for robust assessments of inclusive and hybrid provision and for work linking relational and psychosocial constructs (e.g. belonging, support) to concrete educational outcomes for autistic pupils (Bond et al., 2016; Falkmer et al., 2015; Fayette & Bond, 2018; Keen et al., 2016; G. Lindsay et al., 2016; Preece & Jordan, 2010). In doing so, it aims both to test whether SRC placement confers added benefit relative to other mainstream models and to identify the relational factors most strongly associated with positive outcomes within and across settings, to inform evidence-based practice and policy. In line with these aims, the hypotheses are as follows:

H1: SRC pupils will show greater improvements than M-SRC and N-SRC pupils in:
 H1a. Psychological well-being.
 H1b. Social inclusion and belonging.
 H1c. Perceived support from teachers and peers.
 H1d. Educational progress.
 H1e. Attendance.
H2: Teacher and peer support will predict outcomes alongside placement:
 H2a. SRC placement and high perceived support will predict better outcomes.
 H2b. Perceived support will add predictive value beyond placement type.
H3: Differences between autistic and non-autistic pupils:
 H3a. Autistic pupils will report lower psychological well-being than non-autistic peers.
 H3b. SRC pupils will show a smaller mental health gap relative to non-autistic peers than N-SRC pupils.

## Method

### Design

The study employed a mixed factorial design, with school placement as the between-groups factor and time (baseline vs follow-up) as the within-groups factor. Dependent variables (DVs) included (a) psychological well-being, (b) social inclusion and belonging, (c) perceived support, (d) educational progress, and (e) school attendance and exclusions. A correlational design was also used to assess associations among variables.

### Participants

#### Autistic participants

Participants were recruited from seven suburban mainstream secondary schools in South-East England, with varying socioeconomic profiles (free school meal eligibility ranged from 8.8% to 33%; national average = 24.6%). Five schools had SRCs for autistic students, while two schools had no SRC. Recruitment per school ranged from 8 to 22 autistic participants. Education, Health & Care Plan (EHCP) prevalence was higher in SRC schools (M = 4.92%, SD = 1.17) than in N-SRC schools (M = 2.45%, SD = 0.49), *p* = 0.04 (national average = 2.7%), while SEN Support rates (i.e. those identified as having special educational needs but without an EHCP) did not differ significantly.

Of 266 total participants, 224 took part at the beginning of Year 1 (T1); 172 at the end of Year 1 (T2); 119 at the end of Y2 (T3) and 144 at the end of Year 3 (T4). In all, 119 took part at two or more timepoints, making them eligible for longitudinal analysis. No significant demographic differences were observed between pupils who completed one versus multiple timepoints. At baseline, the majority of autistic participants (*n* = 81) were at the beginning of Year 7 and were therefore newly enrolled in their school provision. A further 23 pupils were in Year 8, indicating 1 year of prior exposure, and 15 pupils were in Year 9, indicating 2 years of exposure prior to baseline. This pattern was broadly similar across placement types. These differences indicate that while most pupils across all groups were new to their placement at baseline, a meaningful minority had already experienced their school environment for 1 or 2 years prior to data collection.

G*Power analysis indicated that for a medium effect size (0.25), alpha = 0.0125 (corrected for multiple measurements) and power = 0.80, 60 participants were required for mixed analyses of variance (ANOVAs) and 123 for regression analyses with six predictors.

All autistic pupils were identified and confirmed as having a formal autism diagnosis by the Heads of Specialist Resource Centres (SRCs) or by SENCos in non-SRC schools; no additional diagnostic verification procedures were undertaken as part of the study. The mean baseline age was 11.85 years (SD = 0.79). Ethnic composition: 106 White, 4 Mixed/Multiple ethnic groups, 4 Asian/Asian British, 4 Black/African/Caribbean/Black British, 1 undisclosed. Table 1 outlines the sample size in each setting type, and statistical comparisons of the main demographic characteristics by group. Apart from EHCP prevalence, demographic differences across placement types were not statistically significant. EHCP status was used as a covariate in relevant analyses.

**Table 1.** Demographic characteristics by school setting and statistical comparisons.

	Setting type			*F/*chi squared/Mann–Whitney *U*	*p*
	SRC (*N* = 50)	M-SRC (*N* = 46)	N-SRC (*N* = 23)		
Gender	M = 39 (78%) *F* = 10 (20%) Non-binary 1 = (2%)	M = 35 (76.1%) *F* = 11 (23.9%) Non-binary = 0 (0%)	M = 12 (52.2%) *F* = 10 (43.5%) Non-binary = 1 (4.3%)	χ^2^ = 6.85 (4)	0.14
Age at baseline	$\bar{x}$ = 143.66 months sd = 10.34	$\bar{x}$ = 140.80 months sd = 8.31	$\bar{x}$ = 141.87 months sd = 9.92	*F* = 1.10 (2, 116)	0.34
Ethnic Background	Asian/Asian British = 2 (4%) Black/African/Caribbean/Black British = 2 (4%) Mixed/Multiple Ethic Groups = 3 (6%) White = 42 (84%) Prefer not to say = 1 (2%)	Asian/Asian British = 2 (4.3%) Black/African/Caribbean/Black British = 2 (4.3%) Mixed/Multiple Ethic Groups = 1 (2.2%) White = 41 (89.1%) Prefer not to say = 0 (0%)	Asian/Asian British = 0 (0%) Black/African/Caribbean/Black British = 0 (0%) Mixed/Multiple Ethic Groups = 0 (0%) White = 23 (100%) Prefer not to say = 0 (0%)	χ^2^ = 4.15 (2) (Based on chi squared with White/Non-White comparison)	0.13
EHCP	Yes = 50 (100%) No = 0 (0%)	Yes = 18 (39.1%) No = 28 (60.9%)	Yes = 7 (30.4%) No = 16 (69.6%)	χ^2^ = 51.09 (2)	<0.001
Co-occurring Conditions	Yes = 31 (63.3%) No = 18 (36.7%)	Yes = 23 (50%) No = 23 (50%)	Yes = 10 (43.5%) No = 13 (56.5%)	χ^2^ = 3.01 (2)	0.22
Pupil Premium	Yes = 9 (18%) No = 41 (82%)	Yes = 9 (19.6%) No = 37 (80.4%)	Yes = 4 (17.4%) No = 19 (82.6%)	χ^2^ = 0.06 (2)	0.97

SRC = specialist resource centres; M-SRC = mainstream placement in SRC host school; N-SRC = mainstream school without an SRC. EHCP = Education, Health & Care Plan.

Across the autistic SRC group, pupils presented with a range of co-occurring needs, most commonly attention deficit hyperactivity disorder (ADHD); sensory processing differences; social, emotional and mental health (SEMH) needs; pathological demand avoidance (PDA); speech, language and communication needs (SLCN); anxiety; dyspraxia and other learning or developmental differences (e.g. dyslexia, global developmental delay). Among autistic pupils in the mainstream of SRC host schools (M-SRC), co-occurring conditions were similarly varied and included ADHD, SEMH, SLCN, language disorders, physical or medical conditions (e.g. renal conditions, scoliosis), developmental coordination difficulties, Fragile X and multi-sensory impairment. Among autistic pupils in non-SRC schools (N-SRC), a smaller number of pupils were reported to have additional needs, most commonly ADHD, anxiety, sensory or motor differences, and other individual needs such as visual/hearing impairments or selective mutism.

#### Non-autistic participants

For comparison with autistic pupils on selected measures of psychological well-being, a comparison group of 119 non-autistic pupils (94 from SRC schools; 25 from N-SRC schools; 75 male, 35 female, 2 non-binary, 7 undisclosed) was recruited from the same schools. Non-autistic pupils were matched on school, gender, year group and (as far as possible) ethnicity. Mean baseline age: 12.44 (SD = 0.81). Ethnic composition: 97 White, 4 Mixed/Multiple ethnic groups, 6 Asian/Asian British, 3 Other, 9 undisclosed.

The study received ethical approval from the University of Surrey Ethics Committee (Ref: FHMS 22-23 161 EGA).

### School types

#### SRC schools

These mainstream secondary schools included dedicated SRCs, designed to support autistic students through specific architectural features like natural lighting, ventilation and quiet areas. Pupils were primarily integrated into mainstream classrooms but had access to the Centre, which offered a structured, low-stimulation space where they could receive tailored support as needed. Staff were trained in autism, and the wider school community received autism awareness training to foster inclusivity.

#### Non-centre schools

N-SRCs had regular SEN provision for pupils with additional needs. One school hosted a Communication and Interaction Needs (COIN) centre, which offered targeted support for a broader group of pupils with communication difficulties, not specifically tailored for autistic learners.

### Procedure

Participants in Years 7 to 9 (ages 11–14) completed surveys at four timepoints: beginning of Year 1 (T1), end of Year 1 (T2), end of Year 2 (T3) and end of Year 3 (T4). Parents and pupils received full information, with parental consent obtained in advance and pupils providing their written consent on the day of participation to conduct and publish the study. Researchers also carefully checked for ongoing assent from pupils throughout the study. Data were collected in familiar, quiet settings, typically in groups of 1 to 10, without discussion. Pupils were informed that their responses would remain anonymous, and they could withdraw at any time. School devices were used to complete *Qualtrics* surveys, supported by staff for reading or comprehension as needed. Average completion time was 12.3 min (range: 5–35 min). In addition, schools provided teacher assessment scores in Mathematics, English, and Science at baseline (T1); the end of Year 1 (T2); and the end of Year 2 (T3).

### Measures

To minimise pupil burden and avoid overly long survey sessions, not all measures were administered at every time point. Instead, we adopted a staggered schedule in which shorter core measures (Internalising Symptoms and Perceived Support) were completed at time points T1, T2 and T3, while the remaining scales were administered at alternating time points (either T1 and T3 or T2 and T4). This design ensured that each construct could still be examined longitudinally across a 2-year period, while keeping the survey length manageable for pupils. Table 2 outlines the measures administered at each time point.

**Table 2.** Overview of data/measures used in analysis by time point.

Measure	T1	T2	T3	T4
Demographics	✓			
Internalising Symptoms	✓	✓	✓	
Peer Support	✓	✓	✓	
Teacher Support	✓	✓	✓	
Academic Self-concept	✓		✓	
Subjective Happiness	✓		✓	
Life Satisfaction	✓		✓	
Flourishing	✓		✓	
Sense of School Belonging		✓		✓
Friendship Satisfaction		✓		✓
Bullying and Victimisation		✓		✓
Teacher Assessments	✓	✓	✓	

(For this study, non-autistic pupils only answered questions from the first four psychological well-being scales below.) For all scales, higher scores reflect greater levels of the construct being measured.

#### Demographics

Collected data included gender, age, ethnic background and three identifier questions for survey matching.

##### (a) Psychological well-being

Internalising symptoms were assessed using the 10 items from the emotional and peer problems subscales of the *Strengths and Difficulties Questionnaire* (SDQ; Goodman, 1997). Items were rated on a 3-point scale (0 = not true, 1 = somewhat true, 2 = certainly true). Subscale scores were calculated by summing the five relevant items (range 0–10) and the total internalising symptoms score by summing both subscales (range 0–20). Internal consistency was high (Cronbach’s α = 0.86).

Subjective happiness was measured using four items from the Subjective Happiness Scale (Lyubomirsky & Lepper, 1999). Items were rated on 7-point scales with response anchors tailored to each statement (e.g. 1 = not a very happy person; 7 = a very happy person), producing a total score between 4 and 28 (α = 0.85).

Life satisfaction was assessed using six items from the *Brief Multidimensional Students’ Life Satisfaction Scale* (Huebner, 1997). Participants rated their satisfaction with key life domains on a 5-point scale (1 = very dissatisfied; 5 = very satisfied), yielding total scores from 6 to 30 (α = 0.83).

Academic self-concept was measured with a 10-item scale developed by Liu and Wang (2005), assessing academic confidence (e.g. ‘I am sure I can learn new things in school’) and academic effort (e.g. ‘I try hard to do well in school’). Items were rated on a 5-point scale (1 = strongly disagree; 5 = strongly agree), with total scores ranging from 10 to 50 (α = 0.88).

Flourishing was assessed using the eight-item *Flourishing Scale* (Diener et al., 2010), which measures psychological well-being indicators such as meaning and purpose. Items were rated on a 5-point scale (1 = strongly disagree; 5 = strongly agree), resulting in total scores from 8 to 40 (α = 0.87).

##### (b) Social inclusion and belonging

Friendship quality was measured using five items from the *Cambridge Hormones and Moods Project Questionnaire* (van Harmelen et al., 2021), assessing aspects such as satisfaction and perceived understanding in friendships. Items were rated on a 5-point scale (1 = very unhappy; 5 = very happy), producing total scores from 5 to 25.

Bullying and victimisation were assessed using the 12-item *Reduced Aggression and Victimisation Scales* (Orpinas & Horne, 2006, as cited in Hamburger et al, 2011), which capture overt and relational victimisation and aggression. Each subscale yields scores from 0 to 36. Reliability was strong for both victimisation (α = 0.89) and bullying (α = 0.87).

Sense of school belonging was measured with the 18-item *Psychological Sense of School Membership* scale (Goodenow, 1993). Items were rated on a 5-point scale, generating total scores from 18 to 90 (α = 0.95).

##### (c) Perceived support

Peer support was assessed using the 10-item peer support subscale of the *Child and Adolescent Social Support Scale* (CASSS; Malecki et al., 2000). Items were rated on a 6-point scale (1 = never; 6 = always), with total scores ranging from 10 to 60 (α = 0.93).

Teacher support was measured using the 10-item teacher support subscale of the CASSS (Malecki et al., 2000), using the same response format as the peer support subscale (α = 0.92).

In addition to the pupil surveys, each school provided data on educational attainment, attendance and exclusions at each time point:

##### (d) Educational attainment

Teacher assessments in Mathematics, English and Science were rated on a 3-point scale (1 = below expected, 2 = expected, 3 = above expected) at T1–T3. Composite scores were averaged per pupil. Data were available for 83 pupils (40 SRC; 33 M-SRC; 10 N-SRC).

##### (e) Attendance and exclusions

Each school reported annual attendance and exclusion data for (i) all pupils, (ii) non-SRC autistic pupils and (iii) SRC pupils. Here, ‘data points’ refer to school-by-time contributions (one school’s records at one wave). Data were averaged across available timepoints, yielding 14 data points for SRC and 6 for N-SRC schools.

### Data analysis

All data were screened prior to analysis to identify missing values, outliers and any violations of normality assumptions. Missing data ranged from 0% to 4.2%. If 25% of items or less were missing, the mean of the remaining scores was imputed. Otherwise pairwise deletion of missing data was implemented. Preliminary checks indicated no violation of normality assumptions: All variables were normally distributed, except the Reduced Aggression and Victimisation Scales. Aggression showed positive skew and leptokurtosis across placements and timepoints. M-SRC Victimisation scores also showed skew and kurtosis. Non-parametric tests were used for these scales. All variables met homogeneity of variance assumptions. Alpha was set at 0.05, with Bonferroni correction applied: adjusted to 0.0125 for both well-being and social inclusion/belonging measures.

Following data cleaning, a series of statistical analyses were conducted to address the study hypotheses. To examine the impact of school placement type and time on autistic pupils’ outcomes, separate 3 (placement type: SRC vs M-SRC vs N-SRC) × 3 (time: T1 vs T2 vs T3) mixed ANOVAs were conducted, separately for each of the first three DVs (Internalising Symptoms, Peer Support, Teacher Support); and separate 3 (placement type: SRC vs M-SRC vs N-SRC) × 2 (time: T1/T2 vs T3/T4) mixed ANOVAs were conducted, separately for each of the remaining DVs, allowing assessment of both main effects and interaction effects across repeated data collection waves. To evaluate predictors of pupil outcomes (H2a–b), hierarchical multiple regressions were performed. Baseline scores for each outcome were entered at the first step to control for initial differences, followed by demographic variables at the second step. Perceived support from teachers and peers was added at the third step, and school placement type – the primary predictor of interest – was entered at the final step, enabling assessment of its unique contribution beyond earlier model components.

To compare autistic and non-autistic pupils on changes in psychological well-being (H3a), an independent samples *t*-test was conducted. Finally, to determine whether well-being differences between autistic and non-autistic pupils varied by school placement type (H3b), two-way ANOVAs were used to test for interaction effects between neurotype and placement.

All analyses were conducted using IBM SPSS Statistics (Version 29).

### Positionality statement

This study was developed within a commitment to participatory and neurodiversity-affirming autism research. The first author is a parent of two autistic young people, bringing long-term lived experience of navigating educational provision, support systems and school inclusion. The second author is AuDHD, contributing an insider perspective on neurodivergent schooling, well-being and the importance of relational and environmental fit. These positions shaped our interest in evaluating SRCs within mainstream schools and in prioritising outcomes that matter to autistic young people and families (e.g. belonging, well-being, perceived support, attendance and attainment).

We recognise that our identities and experiences may influence the questions we ask, the interpretations we privilege and the implications we draw. To strengthen accountability, we worked iteratively with National Autistic Society (NAS) colleagues throughout study design, measure review and interpretation of findings. We also treated participants’ accounts over the three-year period as a source of expertise, using their feedback to refine procedures and enhance accessibility. While we aimed to centre autistic perspectives, we acknowledge that we are not positioned to represent the full diversity of autistic experiences; these findings should therefore be read as situated within UK educational contexts and within ongoing efforts to improve inclusive practice.

## Results

### Psychological well-being, social inclusion and belonging, and perceived support

Contrary to our hypotheses that SRC pupils would show greater improvements than M-SRC and N-SRC pupils in psychological well-being (H1a), social inclusion and belonging (H1b), and perceived support from teachers and peers (H1c), no significant main effects of school placement were found for any outcomes in these domains (see Tables 3 and 4).

**Table 3.** Means (standard deviations) and ANOVA results by school placement (SRC, M-SRC, N-SRC) and time (T1–T4).

Measure (with *n*s)	SRC M (SD)				M-SRC M (SD)				N-SRC M (SD)				Main effect of setting	Main effect of time	Setting × time
	T1	T2	T3	T4	T1	T2	T3	T4	T1	T2	T3	T4			
Teacher Support SRC *n* = 37 M-SRC *n* = 21 N-SRC *n* = 19	42.54 (9.33)	41.16 (10.74)	42.59 (10.24)	—	44.48 (7.68)	40.33 (11.00)	39.81 (11.35)	—	44.16 (7.13)	42.16 (8.18)	42.16 (8.59)	—	*F*(2,73) = 0.45 *p* = 0.64 η_p_^2^ = 0.01	*F*(2,146) = 3.06 *p* = 0.05 η_p_^2^ = 0.04	*F*(4,146) = 0.53 *p* = 0.72 η_p_^2^ = 0.01
Peer Support SRC *n* = 36 M-SRC *n* = 20 N-SRC *n* = 18	33.81 (12.19)	30.97 (11.20)	33.17 (12.87)	—	36.80 (8.33)	34.20 (9.69)	31.95 (10.71)	—	35.83 (9.77)	32.06 (10.57)	29.78 (8.54)	—	*F*(2,70) = 0.24 *p* = 0.78 η_p_^2^ = 0.01	*F*(2,140) = 3.29 *p* = 0.05 η_p_^2^ = 0.05	*F*(2,140) = 0.51 *p* = 0.69 η_p_^2^ = 0.01
I*n*ter*n*alisi*n*g Symptoms (SDQ) SRC *n* = 31 M-SRC *n* = 20 N-SRC *n* = 14	8.23 (3.99)	8.39 (4.49)	7.84 (3.98)	—	7.20 (3.82)	7.50 (4.66)	7.50 (4.29)	—	8.43 (4.01)	8.79 (4.73)	8.79 (4.06)	—	*F*(2,61) = 0.71 *p* = 0.50 η_p_^2^ = 0.02	*F*(2,122) = 2.62 *p* = 0.08 η_p_^2^ = 0.04	*F*(4,122) = 0.45 *p* = 0.77 η_p_^2^ = 0.02
Peer Problems (SDQ) SRC *n* = 31 M-SRC *n* = 20 N-SRC *n* = 14	3.94 (2.17)	3.71 (2.15)	3.58 (2.23)	—	2.80 (1.96)	3.20 (2.35)	3.00 (2.22)	—	3.42 (1.87)	3.86 (2.60)	3.50 (2.25)	—	*F*(2,61) = 1.63 *p* = 0.21 η_p_^2^ = 0.05	*F*(2,122) = 2.12 *p* = 0.12 η_p_^2^ = 0.03	*F*(4,122) = 2.30 *p* = 0.11 η_p_^2^ = 0.04
Emotio*n*al Problems (SDQ) SRC *n* = 31 M-SRC *n* = 20 N-SRC *n* = 14	4.29 (2.56)	4.68 (3.04)	4.26 (2.62)	—	4.40 (2.37)	4.30 (3.03)	4.50 (2.88)	—	5.00 (2.57)	4.93 (2.92)	5.29 (2.34)	—	*F*(2,61) = 0.24 *p* = 0.79 η_p_^2^ = 0.01	*F*(2,122) = 1.28 *p* = 0.28 η_p_^2^ = 0.02	*F*(4,122) = 1.46 *p* = 0.24 η_p_^2^ = 0.02
Academic Self-Co*n*cept SRC *n* = 31 M-SRC *n* = 16 N-SRC *n* = 13	31.90 (7.44)	—	33.26 (6.87)	—	34.88 (8.53)	—	33.38 (7.26)	—	36.69 (5.22)	—	34.23 (5.46)	—	*F*(2,56) = 1.15 *p* = 0.32 η_p_^2^ = 0.04	*F*(1,56) = 2.88 *p* = 0.10 η_p_^2^ = 0.05	*F*(2,56) = 1.93 *p* = 0.17 η_p_^2^ = 0.03
Subjective Happi*n*ess SRC *n* = 30 M-SRC *n* = 16 N-SRC *n* = 13	17.87 (5.99)	—	17.83 (5.56)	—	18.38 (4.73)	—	18.00 (6.43)	—	18.31 (5.04)	—	17.31 (4.72)	—	*F*(2,55) = 0.05 *p* = 0.95 η_p_^2^ = 0.00	*F*(1,55) = 0.64 *p* = 0.43 η_p_^2^ = 0.01	*F*(2,55) = 0.01 *p* = 0.99 η_p_^2^ = 0.00
Life Satisfactio*n* SRC *n* = 31 M-SRC *n* = 16 N-SRC *n* = 13	23.16 (4.15)	—	22.42 (4.21)	—	22.69 (4.25)	—	21.44 (4.43)	—	23.92 (5.22)	—	23.92 (3.80)	—	*F*(2,56) = 1.0 *p* = 0.36 η_p_^2^ = 0.04	*F*(1,56) = 0.26 *p* = 0.61 η_p_^2^ = 0.01	*F*(2,56) = 1.54 *p* = 0.22 η_p_^2^ = 0.03
Flourishi*n*g SRC *n* = 31 M-SRC *n* = 16 N-SRC *n* = 13	28.25 (6.48)	—	28.03 (6.90)	—	30.25 (4.09)	—	27.44 (5.74)	—	31.25 (4.32)	—	31.00 (4.36)	—	*F*(2,56) = 1.95 *p* = 0.15 η_p_^2^ = 0.07	*F*(1,56) = 2.49 *p* = 0.12 η_p_^2^ = 0.04	*F*(2,56) = 0.84 *p* = 0.36 η_p_^2^ = 0.02
Se*n*se of Belo*n*gi*n*g SRC *n* = 32 M-SRC *n* = 31 N-SRC *n* = 12	—	61.66 (16.05)	—	64.28 (14.05)	—	63.97 (12.31)	—	61.23 (11.97)	—	60.67 (14.69)	—	52.42 (16.54)	*F*(2,71) = 0.96 *p* = 0.39 η_p_^2^ = 0.03	*F*(1,71) = 3.71 *p* = 0.06 η_p_^2^ = 0.05	*F*(2,71) = 1.13, *p* = 0.29 η_p_^2^ = 0.02
Frie*n*dship Quality SRC *n* = 29 M-SRC *n* = 32 N-SRC *n* = 12	—	16.24 (4.44)	—	18.97 (3.75)	—	15.31 (5.69)	—	19.37 (4.00)	—	16.75 (4.39)	—	18.08 (3.99)	*F*(2,69) = 0.49 *p* = 0.62 η_p_^2^ = 0.01	*F* **(1,69)** = 7.27* p = 0.01 η_p_^2^ = 0.10	*F*(2,69) = 0.14 *p* = 0.71 η_p_^2^ = 0.00

SRC = specialist resource centre; M-SRC = autistic pupils in mainstream of SRC schools; N-SRC = autistic pupils in non-SRC schools.

* *p* < .0125

**Table 4.** Kruskal–Wallis tests comparing change scores across placement types.

Measure (with *n*s)	SRC Median (IQR)	M-SRC Median (IQR)	N-SRC Median (IQR)	Kruskal–Wallis test
Change in Bullying SRC *n* = 30 M-SRC *n* = 32 N-SRC *n* = 13	0 (−1.75 to 1.00)	0 (−0.75 to 1.00)	0 (−5.00 to 0.50)	χ^2^(2) = 0.78 *p* = 0.68
Change in Victimisation SRC *n* = 31 M-SRC *n* = 32 N-SRC *n* = 13	−1 (−10.00 to 4.00)	0 (−5.50 to 3.00)	2 (14.50 to 3.50)	χ^2^(2) = 1.06 *p* = 0.59

However, Friendship Quality improved significantly over time, *F*(1, 69) = 7.27, *p* = 0.01, partial η^2^ = 0.10. Mean scores indicate a significant increase in friendship quality over time (T1: *x̄* = 15.92, SD = 4.99; T3: *x̄* = 18.98, SD = 4.14).

### Educational progress

**Figure 1.**
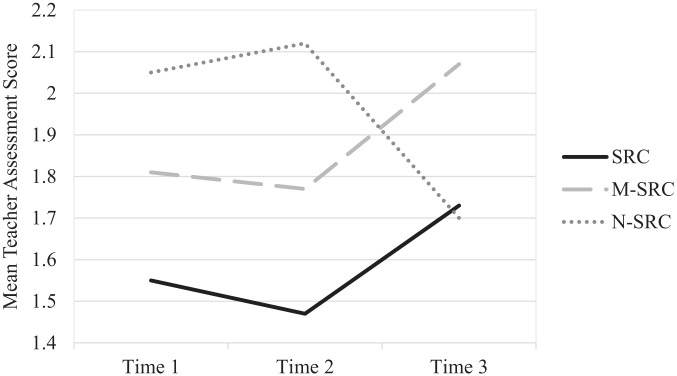
Academic progression in core subjects according to placement and time. SRC = specialist resource centres; M-SRC = mainstream placement in SRC host school; N-SRC = mainstream school without an SRC.

In relation to our hypothesis that SRC pupils would show greater educational progress than M-SRC and N-SRC pupils (H1d), analysis of attainment revealed a significant main effect of placement, *F*(2, 81) = 4.42, *p* = 0.015, partial η^2^ = 0.10, indicating differences in attainment across settings within this sample. However, this must be interpreted cautiously due to a significant Placement × Time interaction, *F*(4, 160) = 3.92, *p* = 0.005, partial η^2^ = 0.09, and the relatively small group sizes for some placements, particularly the N-SRC group. Figure 1 shows that N-SRC pupils declined from Time 2 to Time 3, while SRC and M-SRC groups improved.

### School attendance

Attendance data over three years showed an average of 89.21% in SRC schools. SRC pupil attendance averaged 87.42%, compared to 86.53% for M-SRC and 85.84% for N-SRC pupils. (These attendance figures were derived from school-level summary data rather than individual-level longitudinal records.) National averages during this period were 91.2% (all pupils) and 86.8% (autism primary need); county-level averages (across the counties included in this study) were 90.9% (all pupils) and 86.2% (autism primary need), respectively.

**Figure 2.**
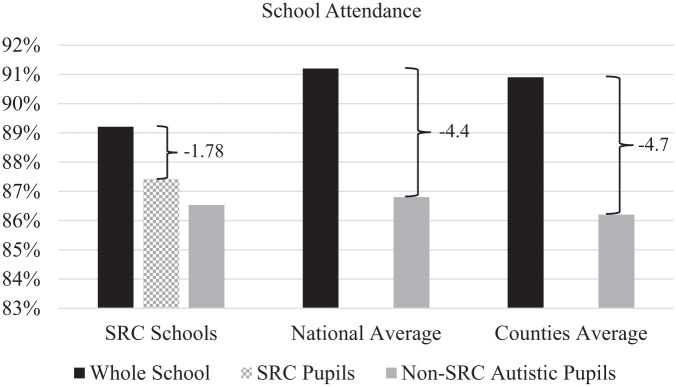
School attendance rates in SRC schools compared with national and county averages.

Whole school and autistic pupil attendance did not differ significantly between SRC and N-SRC schools. However, when examining attendance disparities (i.e. the difference between subgroup attendance and whole school attendance), SRC pupils had a smaller attendance gap relative to their school average (*x̄* = −1.78) than the national gap observed between all pupils and those with autism as a primary need (*x̄* = −4.4). This difference was statistically significant, *t*(13) = 2.68, *p* = 0.01 (one-tailed), with a medium-large effect size, *d* = 0.72, 95% confidence interval (CI): [0.50, 4.73] (see Figure 2).

Similarly, the disparity between SRC pupil attendance and whole school attendance was significantly smaller than the disparity between county-level whole school attendance and autism primary need attendance (*x̄* = −4.7), *t*(13) = 2.98, *p* = 0.005 (one-tailed), with a large effect size, *d* = 0.80, 95% CI: [0.18, 1.39]. These comparisons rely on aggregated data and limited *N*s and therefore provide indicative rather than definitive evidence.

### School exclusion

No significant group differences were found in exclusion outcomes; however, some descriptive patterns were evident. When examining exclusion data relative to all exclusions within each school type, SRC pupils accounted for 3% of fixed-term exclusions in their schools. In comparison, M-SRC pupils accounted for 5% of exclusions, and N-SRC pupils accounted for 4%. A similar pattern was observed for exclusion days: SRC pupils accounted for 3% of all days lost to exclusion in their schools, whereas M-SRC and N-SRC pupils each accounted for 6%. Given the low overall number of exclusions and small subgroup sizes, these patterns should be interpreted cautiously.

### Associations with psychological, social and educational outcomes

Placement group alone had limited impact on changes in social and psychological well-being outcomes, as indicated by non-significant effects in the ANOVAs, suggesting that placement type was not a primary driver of change across most outcomes. However, given the quasi-experimental design, uneven and/or modest group sizes for some outcomes, null placement effects should be treated cautiously because smaller effects may have gone undetected. To better describe patterns within the data, we therefore explored individual-level associations using regression analyses. The regression analyses, while identifying associations with follow-up outcomes after accounting for baseline (i.e. residualised change), were exploratory in nature and do not establish causality. Because perceived support and outcomes were measured at the same time, directionality cannot be inferred, and bidirectional or unmeasured influences are possible; these findings should therefore be interpreted as correlates rather than definitive drivers of change. To examine factors associated with follow-up scores for each of the outcome variables, hierarchical regressions were run with four steps: (1) Baseline score, (2) Demographic variables (age, gender), (3) Perceived Teacher Support and Perceived Peer Support and (4) Placement group (SRC, M-SRC, N-SRC). (See Supplementary Materials (Tables 9–30) for full correlation matrices and hierarchical regression results.)

#### Perceived peer support

*Perceived peer support* showed the most consistent and robust associations with outcomes across the analysis. Higher perceived peer support was most strongly associated with decreased internalising symptoms (β = –0.442, *p* < 0.001), fewer peer problems (β = –0.653, *p* < 0.001), greater subjective happiness (β = 0.404, *p* = 0.002) and greater friendship quality (β = 0.524, *p* < 0.001). Perceived peer support accounted for substantial variance within models: internalising symptoms: 63.8% of the variance, *F*(7, 74) = 18.59, *p* < 0.001; peer problems: 60%, *F*(7, 74) = 15.71, *p* < 0.001); subjective happiness: 44.1%, *F*(7, 51) = 5.74, *p* < 0.001; friendship quality: 38.3%, *F*(7, 63) = 5.68, *p* < 0.001. Perceived peer support was also significantly associated with higher levels of flourishing (β = 0.329, *p* = 0.007) and stronger school belonging (β = 0.259, *p* = 0.001).

#### Perceived teacher support

*Perceived teacher support* was also significantly associated with several outcomes, being the factor most strongly associated with pupils’ sense of school belonging (β = 0.466, *p* < 0.001) and increased flourishing (β = 0.375, *p* = 0.004). In the school-belonging model, perceived teacher support accounted for 75.1% of the variance, *F*(7, 63) = 27.08, *p* < 0.001, while it explained 49.5% of the variance in flourishing, *F*(7, 52) = 7.27, *p* < 0.001. Perceived teacher support was also associated with fewer peer problems (β = 0.229, *p* = 0.01).

#### Gender

*Gender* was a significant demographic associated with some outcomes. Being female was associated with more internalising symptoms (β = 0.274, *p* < 0.001) and was the only association with greater emotional problems (β = 0.331, *p* < 0.001). Emotional problems were explained with 59.1% of the variance, *F*(7, 74) = 15.28, *p* < 0.001.

#### Placement in an SRC

*Placement in an SRC* was associated with higher academic attainment compared to N-SRC placement (β = −0.229, *p* = 0.007), but lower academic attainment compared to M-SRC placement (β = 0.183, *p* = 0.03). Placement accounted for 23% of the variance in academic attainment, *F*(5,116) = 6.94, *p* < 0.001. SRC placement was also associated with higher perceived teacher support when compared to M-SRC (β = –0.207, *p* = 0.02), and with stronger school belonging than placement in non-SRC schools (β = –0.295, *p* < 0.001).

No predictors emerged for academic self-concept, life satisfaction, bullying or victimisation.

### Differences in psychological well-being of autistic and non-autistic pupils

A series of 2 × 2 ANOVAs were conducted to examine change scores (follow-up minus baseline) for autistic and non-autistic pupils in SRC and N-SRC schools. Due to non-normality, Mann–Whitney U tests were used for some variables. Contrary to our hypotheses that autistic pupils would report lower psychological well-being than non-autistic peers (H3a) and that SRC pupils would show a smaller well-being gap than those in N-SRC schools (H3b), placement type did not show main effects and no significant differences were found in internalising symptoms (Table 5), subjective happiness, life satisfaction or academic self-concept (Table 6).

**Table 5.** Means (standard deviations) and ANOVA results by school setting (SRC school vs N-SRC school) and neurotype (autistic vs non-autistic).

Measure (with *n*s)	ASC SRCS M (SD)	ASC N-SRCS M (SD)	N-ASC SRCS M (SD)	N-ASC N-SRCS M (SD)	Main effect: setting	Main effect: neurotype	Setting × neurotype
Change in Internalising Symptoms ASC *n* = 83 N-ASC *n* = 116	−0.12 (3.63)	0.33 (2.82)	1.54 (3.76)	1.08 (3.76)	*F* = 0.06 *p* = 0.80 η_p_^2^ < 0.001	*F* = 1.08 *p* = 0.30 η_p_^2^ = 0.006	*F* = 0.17 *p* = 0.69 η_p_^2^ = 0.001
Change in Peer Problems ASC *n* = 83 N-ASC *n* = 116	−0.19 (2.15)	0.07 (2.15)	0.65 (1.94)	0.42 (1.74)	*F* = 0.19 *p* = 0.67 η_p_^2^ = 0.001	*F* = 0.24 *p* = 0.63 η_p_^2^ = 0.001	*F* = 0.03 *p* = 0.85 η_p_^2^ = 0.000
Change in Emotional Problems ASC *n* = 83 N-ASC *n* = 116	0.07 (2.23)	0.27 (1.79)	0.89 (2.44)	0.67 (2.51)	*F* = 0.001 *p* = 0.98 η_p_^2^ = 0.000	*F* = 1.45 *p* = 0.23 η_p_^2^ = 0.007	*F* = 0.23 *p* = 0.64 η_p_^2^ = 0.001

SRCS = specialist resource centre school; N-SRCS = non-SRC school; ASC = autism spectrum condition; N-ASC = non-autistic.

**Table 6.** Comparison of change scores between autistic and non-autistic pupils in both school types.

Measure	ASC *n*	ASC median (IQR)	N-ASC *n*	N-ASC median (IQR)	*U*	*p*
Change in Subjective Happiness	60	0 (−3 to 3)	111	−1 (−5 to 1)	2271	0.07
Change in Life Satisfaction	61	0 (−3.5 to 1)	117	0 (−4 to 1)	3455.5	0.73
Change in Academic Self-Concept	61	1 (−5 to 5)	116	−2 (−6 to 3)	3075	0.15

However, these analyses combined all autistic pupils (both SRC and M-SRC) within SRC schools. To explore the specific impact of the SRCs, we conducted additional exploratory analyses comparing SRC pupils to non-autistic peers from the same schools. An independent samples *t*-test revealed a significant group difference in change scores for internalising symptoms. SRC pupils showed a decrease in internalising symptoms, while non-autistic pupils showed an increase. Further analysis of the internalising subscales showed that this effect was primarily driven by changes in peer problems. A significant group difference was observed in peer problems change scores. SRC pupils showed a reduction in peer problems, whereas non-autistic pupils reported an increase. No significant difference was found for the emotional problems subscale (see Table 7).

**Table 7.** Comparison of change scores between autistic and non-autistic pupils in SRC schools only.

Measure	*n* (autistic)	*n* (non-autistic)	Autistic M (SD)	Non-autistic M (SD)	Test statistic	*p*	*d*
Change in Internalising Symptoms	36	92	−0.03 (3.76)	1.54 (3.76)	*t* **(126)** = −**2.13**	0.04*	−**0.42**
Change in Peer Problems	36	92	−0.28 (2.02)	0.65 (1.94)	*t* **(126)** = **2.41**	0.02*	−**0.47**
Change in Emotional Problems	36	92	0.25 (2.40)	0.89 (2.44)	*t*(126) = −1.24	0.18	−0.26

* *p* < .05

The only other significant finding when comparing autistic and non-autistic pupils from SRC schools was in changes in subjective happiness. A Mann–Whitney *U* test indicated that SRC pupils’ subjective happiness remained stable (median = 0.00, *n* = 31), whereas non-autistic pupils experienced a decline (median = −2.00, *n* = 88). This difference was small and approached statistical significance, *U* = 1043.5, *z* = −1.95, *p* = 0.05, *r* = 0.18 (see Table 8).

**Table 8.** Comparison of change scores between autistic and non-autistic pupils in SRC schools only.

Measure	*n* (autistic)	Autistic Median (IQR)	*n* (non-autistic)	SRC non-autistic Median (IQR)	*U*	*p*
Change in Subjective Happiness	31	0.00 (–4.00 to 3.00)	88	–2.00 (–5.00 to 1.00)	1043.50	0.05
Change in Life Satisfaction	32	0.00 (–3.00 to 1.75)	93	0.00 (–4.00 to 1.00)	1372.50	0.51
Change in Academic Self-Concept	32	2.00 (–3.00 to 5.00)	93	–1.00 (–5.50 to 3.00)	1170.00	0.07

## Discussion

This study evaluated whether autistic pupils’ outcomes differed according to placement in specialist resource centres (SRCs), mainstream placements in schools with SRCs (M-SRC) or mainstream schools without SRCs but with other SEN provision (N-SRCs). Across psychological well-being, social inclusion, educational progress, attendance, exclusion and perceived support, the overall pattern was mixed: placement type showed limited direct association with change in most psychosocial outcomes, yet SRC placement was linked to specific advantages in school-experience and educational domains. At the same time, perceived peer and teacher support emerged as having the most consistent associations with positive adjustment across settings. This pattern aligns with international research and policy frameworks, emphasising that inclusive education depends less on placement labels and more on everyday participation, equity and relational fit within schools (Ainscow, 2020; García-Cedillo et al., 2015; Ito et al., 2023; Pfeffer, 2015; UNESCO, 2009).

Contrary to hypotheses that SRC placement would produce stronger psychosocial gains than M-SRC or N-SRC placements, mixed ANOVAs did not detect significant placement-related differences in trajectories for most well-being, social or perceived support outcomes. These null findings are important in their own right. They echo qualitative and review evidence showing that autistic pupils’ mainstream experiences are highly heterogeneous, with outcomes shaped by the quality of provision rather than the presence or absence of a specific placement model (Aubineau & Blicharska, 2020; Bond et al., 2016; Goodall, 2018a, 2018b; Hill, 2014; Horgan et al., 2022; Humphrey & Lewis, 2008; Poon et al., 2014; Richter et al., 2020; Saggers, 2015). The absence of detectable placement differences may therefore reflect substantial within-group variability in inclusive practice, staffing stability or school climate, all of which are known to influence autistic pupils’ adjustment (Falkmer et al., 2015; Krieger et al., 2018; S. Lindsay et al., 2013; McAllister & Sloan, 2016; Neal & Frederickson, 2016). It may also indicate that when mainstream schools develop strong autism-inclusive approaches, psychosocial outcomes can be comparable across placement types (Bond & Hebron, 2016; Croydon et al., 2019; Hebron & Bond, 2017; Keane et al., 2012; Roberts et al., 2008).

At the same time, regression analyses suggested that SRC placement was linked to stronger school belonging and higher perceived teacher support compared with some mainstream placements once baseline and covariates were controlled. This is consistent with prior work describing SRCs and satellite models as potentially providing calmer, more predictable spaces and specialist expertise that can enhance emotional safety and adult–pupil trust (Bond & Hebron, 2016; Croydon et al., 2019; Goodall, 2019; Hebron & Bond, 2017; McAllister & Sloan, 2016; Tobias, 2009). These associations also fit broader belonging frameworks which highlight the role of safe relationships and feeling ‘known’ in school (Carter, 2021; Shochet et al., 2006). Nevertheless, given non-random placement and the inability to model school-level factors, these results should be interpreted as associations that may partly reflect contextual differences between schools.

Educational outcomes were similarly nuanced. Attainment improved over time among SRC and M-SRC pupils but declined for N-SRC pupils, and regressions indicated SRC pupils outperformed N-SRC peers but underperformed relative to M-SRC peers. This mirrors literature suggesting that academic outcomes for autistic pupils are tied to systemic features such as teacher training, resourcing and inclusive infrastructure, rather than placement form alone (Horan & Merrigan, 2019; Keen et al., 2016; Kurth & Mastergeorge, 2010; G. Lindsay et al., 2016; Robertson et al., 2003). It also aligns with evidence that autistic pupils can succeed academically in mainstream settings when environmental demands are well matched but may struggle when support is inconsistent or sensory/social stressors are unaddressed (Ashburner et al., 2010; Goodall, 2018a; Krieger et al., 2018). The decline in N-SRC attainment and the advantage for SRC/M-SRC pupils may therefore reflect differences in whole-school SEN capacity rather than an effect unique to SRC placement. Nevertheless, because placement decisions are shaped by need, advocacy and local availability, and attainment sample sizes were modest, causal claims are not warranted.

Attendance and exclusions provide tentative but policy-relevant signals. SRC pupils’ attendance moved closer to whole-school averages and was more favourable than national/local benchmarks for autistic pupils, suggesting a possible attendance-equity benefit. This resonates with emerging evidence that school distress and unmet need drive autistic non-attendance, and that environments offering predictability and reduced sensory/social load may reduce barriers to attendance (E. Chapman, 2023; Connolly et al., 2023; Goodall, 2019; Krieger et al., 2018). Exclusion patterns descriptively favoured SRC pupils but were non-significant. This is directionally consistent with research showing autistic pupils’ disproportionate exclusion risk and the downstream harms of exclusion, but the present data remain underpowered for firm conclusions. These trends warrant replication in larger cohorts with school-level modelling.

The most robust and consistent finding was that perceived peer support and, to a lesser extent, perceived teacher support were strongly associated with positive outcomes across multiple domains. Higher peer support was associated with fewer internalising symptoms and peer problems and higher subjective happiness, friendship quality, flourishing and school belonging. These effects replicate and extend a well-established literature showing that peer acceptance, friendship quality and supportive classroom climates are protective for autistic pupils’ mental health and school engagement (Bauminger & Kasari, 2000; Chamberlain et al., 2007; Cook et al., 2016, 2018; Cresswell et al., 2019; Humphrey & Symes, 2010; Kasari et al., 2011; Locke et al., 2010; Newcomb & Bagwell, 1995; Petrina et al., 2014). They also speak directly to bullying and social vulnerability research in autism, where low peer support is repeatedly linked to victimisation and poorer adjustment (Carrington et al., 2017; Cook et al., 2020; Hebron et al., 2015; Maiano et al., 2016).

Perceived teacher support uniquely contributed to flourishing and belonging and was linked to fewer peer problems. This aligns with broader evidence that affective teacher–student relationships buffer against anxiety and disengagement and support academic/social motivation (Jennings & Greenberg, 2009; Robertson et al., 2003; Roorda et al., 2011; Sakiz et al., 2012; Shechtman & Leichtentritt, 2004; Wentzel et al., 2010). This study extends these relational findings by demonstrating their strength across multiple outcomes within a relatively large, multi-site, longitudinal cohort spanning different mainstream-embedded placements, while also accounting for baseline functioning and demographics.

These associations also align with existing qualitative accounts from autistic pupils’ which emphasise the importance of predictability, sensory safety and acceptance to participate socially and emotionally in mainstream schools (Aubineau & Blicharska, 2020; Billington et al., 2024; Goodall, 2018a, 2018b, 2019; Humphrey & Lewis, 2008). They also intersect with work on stigma and masking: if social environments require autistic pupils to camouflage, this may erode well-being and distort perceived support, particularly for girls (Botha et al., 2022; L. Chapman et al., 2022; Dean et al., 2017; den Houting et al., 2021; Halsall et al., 2021; Hull et al., 2020; Mandy et al., 2012; Solomon et al., 2012; Tierney et al., 2016). Future work should test these mechanisms explicitly using mediation and cross-lagged models and richer measurement of school-level variables.

The absence of significant differences in *change* between autistic and non-autistic pupils across placements contrasts with the wider evidence base showing elevated rate of anxiety, depression and internalising symptoms in autistic adolescents (Avenevoli et al., 2015; Greenlee et al., 2016; Hebron & Humphrey, 2014; Kim et al., 2000; Lai et al., 2019; Mayes et al., 2013; Merikangas et al., 2010; Mertens et al., 2017; Van Steensel et al., 2011). One interpretation is that participating schools provided comparatively protective environments, narrowing typical gaps. Another is that limited power for some outcomes or selective attrition constrained detection of differences. The pattern of stability or improvement for autistic pupils within SRC contexts is consistent with the view that supportive environments can mitigate risk, but stronger causal designs are needed to establish this.

### Implications

These results suggest that policy decisions focused solely on placement will be incomplete unless they also address the relational and environmental conditions that enable inclusion. This echoes inclusive education scholarship stressing that equitable outcomes depend on school culture, relationships and the removal of participation barriers, not simply on location (Ainscow, 2020; Norwich, 2007; Pfeffer, 2015; UNESCO, 1994, 2009; Warnock, 2005). Schools across all placement types should prioritise peer-mediated approaches, explicit relationship-building and staff development that support relational inclusion and autism-affirming practice (Falkmer et al., 2015; Jennings & Greenberg, 2009; Krieger et al., 2018; S. Lindsay et al., 2013; Wentzel et al., 2010). SRC schools may provide structural advantages – sensory adaptation, predictability, specialist staffing – but these features may matter primarily through their potential to support relationships and reduce stressors that undermine belonging and engagement (Goodall, 2019; Hebron & Bond, 2017; McAllister & Sloan, 2016).

In summary, placement group alone was not a consistent predictor of psychosocial change, so claims about SRC ‘benefits’ should remain tightly bounded to the domains where associations were observed (belonging, perceived teacher support, attainment trajectories and possibly attendance trends). The strongest contribution of the study is its clear multi-outcome evidence that perceived peer and teacher support are central correlates of adjustment across school contexts, extending a strong existing literature through a robust longitudinal, multi-site design. Understanding and testing the mechanisms that generate these relational experiences – including sensory adaptation, predictability, stigma reduction and structured opportunities for peer connection – should be central to future research and inclusive practice.

### Limitations

Several limitations should be acknowledged. First, the quasi-experimental design, with non-random placement into SRC, M-SRC and N-SRC provision, limits causal inference; findings should be interpreted as associations rather than effects of placement. Although schools were matched on key demographics, school-level factors that were not measured or modelled – such as leadership priorities, ethos, resourcing, staff turnover, inclusion policies and wider local authority commissioning practices – may systematically differ by setting type and could have shaped pupils’ trajectories. Because the number of participating schools was modest, it was not possible to model school as a higher-level (random) factor, so these contextual influences cannot be disentangled from placement effects.

Second, selection effects are likely: placement decisions typically reflect a mix of pupil need, family advocacy, professional judgement and local availability of provision. SRC-placed pupils may therefore enter with greater complexity or different profiles of need than peers in mainstream settings. Even with EHCP status statistically controlled, residual differences in severity or co-occurring difficulties may have reduced comparability between groups and masked placement-related benefits.

Third, group sizes were uneven, and the N-SRC cohort was relatively small, limiting statistical power and precision for detecting setting differences and interactions over time. This increases the risk of Type II errors and means that null findings – particularly for N-SRC comparisons – should be interpreted cautiously. In addition, several of the hierarchical regression models were likely underpowered relative to the a priori G*Power estimate for regressions with six predictors, especially where outcome *N*s were reduced by missing data or where effects were small. As a result, non-significant predictors in these models may reflect limited power rather than true absence of association, and the pattern of significant findings should be interpreted cautiously.

Fourth, although most participants began the study at the start of Year 7 and were therefore new to their school provision, a proportion had already been in their placement for 1 or 2 years. This distribution was broadly similar across placement types, though the SRC group included slightly more pupils with prior exposure. Such differences may have influenced both baseline functioning and the magnitude of change detectable over time, as pupils already settled into their provision may have experienced benefits (or challenges) prior to data collection. Future work should measure duration and intensity of placement more systematically to better isolate school-type effects.

Fifth, parent survey data were not included in the present analyses. Parent-reported data can provide an important additional perspective and strengthen triangulation alongside pupil self-report. Parent questionnaires were initially collected at early time points but experienced substantial attrition, resulting in insufficient and potentially biased data for robust quantitative or longitudinal analysis. The absence of parent-report data therefore represents a limitation, and future research would benefit from incorporating sustained parent perspectives alongside pupil and school-reported measures.

Finally, while self-report measures are vital for capturing pupil perspectives, they are susceptible to social desirability bias and may miss subtle but meaningful changes in emotional well-being or perceived support. Perceived teacher and peer support were also measured at the same time as follow-up outcomes, so the regressions cannot establish whether support drove later adjustment or whether pupils’ adjustment shaped how supported they felt (i.e. bidirectionality). Longer-lag or cross-lagged designs are needed to clarify directionality.

### Future directions

Future research should explore the mechanisms behind the effects of SRCs, or similar models, integrating quantitative and qualitative data. It should also examine how perceived support affects outcomes and clarify whether these relational dynamics mediate the observed outcomes, potentially through pathways such as reduced anxiety, greater school belonging or increased motivation. Understanding how these social relationships are fostered (or hindered) may offer valuable guidance for broader inclusive practice. In addition, the integration of individual-level data, such as attendance and exclusion patterns, could provide a more nuanced picture of who benefits most from this type of provision. This may help to identify key student characteristics, such as profile of need, level of support or prior school experience, that are most compatible with the SRC model, offering a clearer understanding of the conditions under which such placements are most successful. Finally, longitudinal studies tracking outcomes beyond secondary school would clarify whether benefits persist into adulthood.

## Conclusion

This study provides important new insights into the educational experiences of autistic pupils across different mainstream school placements. While specialist resource centres were not universally associated with psychosocial advantages, they were linked to better academic progress, stronger sense of belonging, attendance equity, higher perceived teacher support and reduced peer difficulties. However, the clearest insight is that supportive relationships are the most powerful drivers of positive outcomes. Educational policy and practice must focus not just on where pupils are placed, but how well they are supported. Creating inclusive school cultures grounded in emotional safety, teacher trust and peer connection will likely yield the most meaningful change. Ultimately, improving outcomes for autistic pupils may not hinge on *where* they are educated, but *how* they are supported – by peers, teachers and the whole school culture.

## Supplemental Material

sj-pdf-1-aut-10.1177_13623613261426099 – Supplemental material for The impact of specialist resource centres on autistic pupils’ experience of mainstream school Supplemental material, sj-pdf-1-aut-10.1177_13623613261426099 for The impact of specialist resource centres on autistic pupils’ experience of mainstream school by Anna Cook and Alice Boddy in Autism

## References

[bibr1-13623613261426099] Ainscow M. (2020). Promoting inclusion and equity in education: Lessons from international experiences. Nordic Journal of Studies in Educational Policy, 6(1), 7–16. 10.1080/20020317.2020.1729587

[bibr2-13623613261426099] Aitken D. Wang L. (2019). Learning difficulties and exclusion from school, Salvesen mindroom research briefing, number 1. https://salvesen-research.ed.ac.uk/sites/default/files/2022-10/Briefing%201%20-%20Learning%20difficulties%20and%20exclusion.pdf

[bibr3-13623613261426099] Ashburner J. Ziviani J. Rodger S. (2010). Surviving in the mainstream: Capacity of children with autism spectrum disorders to perform academically and regulate their emotions and behavior at school. Research in Autism Spectrum Disorders, 4(1), 18–27. 10.1016/j.rasd.2009.07.002

[bibr4-13623613261426099] Aubineau M. Blicharska T. (2020). High-functioning autistic students speak about their experience of inclusion in mainstream secondary schools. School Mental Health, 12(3), 537–555. 10.1007/s12310-020-09364-z

[bibr5-13623613261426099] Avenevoli S. Swendsen J. He J. P. Burstein M. Merikangas K. R. (2015). Major depression in the national comorbidity survey-adolescent supplement: Prevalence, correlates, and treatment. Journal of the American Academy of Child & Adolescent Psychiatry, 54(1), 37–44. 10.1016/j.jaac.2014.10.010 25524788 PMC4408277

[bibr6-13623613261426099] Bauminger N. Kasari C. (2000). Loneliness and friendship in high-functioning children with autism. Child Development, 71(2), 447–456. 10.1111/1467-8624.00156 10834476

[bibr7-13623613261426099] Boddy A. Cook A. (2026). Specialist resource centres as protective microsystems: A qualitative comparative case study of Autistic Pupils’ experiences in mainstream secondary schools. [Preprint] PsyArXiv. 10.31234/osf.io/h8zgq_v1 PMC13392175 42389848

[bibr8-13623613261426099] Billington J. Loucas T. Knott F. (2024). ‘I liked school, but school didn’t like me’: Autistic young adults’ reflections on their mainstream primary school experiences. Neurodiversity, 2. 10.1177/27546330241310174

[bibr9-13623613261426099] Bond C. Hebron J. (2016). Developing mainstream resource provision for pupils with autism spectrum disorder: Staff perceptions and satisfaction. European Journal of Special Needs Education, 31(2), 250–263. 10.1080/08856257.2016.1141543

[bibr10-13623613261426099] Bond C. Symes W. Hebron J. Humphrey N. Morewood G. (2016). Educating persons with autistic spectrum disorder-A systematic literature review. The National Council for Special Education [NCSE], 20, 1–246.

[bibr11-13623613261426099] Botha M. Dibb B. Frost D. M. (2022). ‘It’s being a part of a grand tradition, a grand counter-culture which involves communities’: A qualitative investigation of autistic community connectedness. Autism, 26(8), 2151–2164. 10.1177/13623613221080248 35318862 PMC9597163

[bibr12-13623613261426099] Brede J. Remington A. Kenny L. Warren K. Pellicano E. (2017). Excluded from school: Autistic students’ experiences of school exclusion and subsequent re-integration into school. Autism & Developmental Language Impairments, 2. 10.1177/2396941517737511

[bibr13-13623613261426099] Carrington S. Campbell M. Saggers B. Ashburner J. Vicig F. Dillon-Wallace J. Hwang Y. S. (2017). Recommendations of school students with autism spectrum disorder and their parents in regard to bullying and cyberbullying prevention and intervention. International Journal of Inclusive Education, 21(10), 1045–1064. 10.1080/13603116.2017.1331381

[bibr14-13623613261426099] Carter E. W. (2021). Dimensions of belonging for individuals with intellectual and developmental disabilities. In Belonging and resilience in individuals with developmental disabilities (pp. 13–34). Springer. 10.1007/978-3-030-81277-5_2

[bibr15-13623613261426099] Chamberlain B. Kasari C. Rotheram-Fuller E. (2007). Involvement or isolation? The social networks of children with autism in regular classrooms. Journal of Autism and Developmental Disorders, 37, 230–242. 10.1007/s10803-006-0164-4 16855874

[bibr16-13623613261426099] Chapman E. (2023). Preventing unmet need from leading to school exclusion: Empowering schools to identify neurodiversity earlier [Doctoral dissertation]. University of Leeds.

[bibr17-13623613261426099] Chapman L. Rose K. Hull L. Mandy W. (2022). ‘I want to fit in. . . but I don’t want to change myself fundamentally’: A qualitative exploration of the relationship between masking and mental health for autistic teenagers. Research in Autism Spectrum Disorders, 99, 102069. 10.1016/j.rasd.2022.102069

[bibr18-13623613261426099] Connolly S. E. Constable H. L. Mullally S. L. (2023). School distress and the school attendance crisis: A story dominated by neurodivergence and unmet need. Frontiers in Psychiatry, 14, Article 1237052. 10.3389/fpsyt.2023.1237052 PMC10556686 37810599

[bibr19-13623613261426099] Cook A. Ogden J. Winstone N. (2016). The experiences of learning, friendship and bullying of boys with autism in mainstream and special settings: A qualitative study. British Journal of Special Education, 43(3), 250–271. 10.1111/1467-8578.12143

[bibr20-13623613261426099] Cook A. Ogden J. Winstone N. (2018). Friendship motivations, challenges and the role of masking for girls with autism in contrasting school settings. European Journal of Special Needs Education, 33(3), 302–315. 10.1080/08856257.2017.1312797

[bibr21-13623613261426099] Cook A. Ogden J. Winstone N. (2020). The effect of school exposure and personal contact on attitudes towards bullying and autism in schools: A cohort study with a control group. Autism, 24(8), 2178–2189. 10.1177/1362361320937088 32668954 PMC7549291

[bibr22-13623613261426099] Cresswell L. Hinch R. Cage E. (2019). The experiences of peer relationships amongst autistic adolescents: A systematic review of the qualitative evidence. Research in Autism Spectrum Disorders, 61, 45–60. 10.1016/j.rasd.2019.01.003

[bibr23-13623613261426099] Croydon A. Remington A. Kenny L. Pellicano E. (2019). ‘This is what we’ve always wanted’: Perspectives on young autistic people’s transition from special school to mainstream satellite classes. Autism & Developmental Language Impairments, 4, 2396941519886475. 10.1177/2396941519886475

[bibr24-13623613261426099] Dean M. Harwood R. Kasari C. (2017). The art of camouflage: Gender differences in the social behaviors of girls and boys with autism spectrum disorder. Autism, 21(6), 678–689. 10.1177/1362361316671845 27899709

[bibr25-13623613261426099] den Houting J. Botha M. Cage E. Jones D. R. Kim S. Y . (2021). Shifting stigma about autistic young people. The Lancet Child & Adolescent Health, 5(12), 839–841. 10.1016/S2352-4642(21)00309-6 34600630

[bibr26-13623613261426099] Department for Education. (2022). SEND review: Right support, right place, right time [Green paper]. https://www.gov.uk/government/consultations/send-review-right-support-right-place-right-time

[bibr27-13623613261426099] Department for Education. (2023). SEND and alternative provision improvement plan. https://www.gov.uk/government/publications/send-and-alternative-provision-improvement-plan

[bibr28-13623613261426099] Department for Education. (2024a, October). Delivering better value in SEND: Phase 1 insight summary. https://cdn.prod.website-files.com/63b6e5debb4b0114060dc226/66421eaae18cb50ccc378780_66421a046d5569ec0ad11674_DBV%20-%20Phase%201%20Insights%20Summary_Website%20v1.0_Final.pdf

[bibr29-13623613261426099] Department for Education. (2024b). Special educational needs in England: Academic year 2023/24. Explore Education Statistics. https://explore-education-statistics.service.gov.uk/find-statistics/special-educational-needs-in-england/2023-24

[bibr30-13623613261426099] Department for Education & Department of Health and Social Care. (2014). SEND code of practice: 0 to 25 years. https://www.gov.uk/government/publications/send-code-of-practice-0-to-25

[bibr31-13623613261426099] Department for Education & Office for National Statistics. (2017). Permanent and fixed period exclusions in England: 2015 to 2016 Education Act 1981, c. 60. (1981). Legislation.gov.uk. https://www.legislation.gov.uk/ukpga/1981/60/enacted

[bibr32-13623613261426099] Diener E. Wirtz D. Tov W. Kim-Prieto C. Choi D. W. Oishi S. Biswas-Diener R. (2010). New well-being measures: Short scales to assess flourishing and positive and negative feelings. Social Indicators Research, 97(2), 143–156. 10.1007/s11205-009-9493-y

[bibr33-13623613261426099] Falkmer M. Anderson K. Joosten A. Falkmer T. (2015). Parents’ perspectives on inclusive schools for children with autism spectrum conditions. International Journal of Disability, Development and Education, 62(1), 1–23. 10.1080/1034912X.2014.984589

[bibr34-13623613261426099] Fayette R. Bond C. (2018). A systematic literature review of qualitative research methods for eliciting the views of young people with ASD about their educational experiences. European Journal of Special Needs Education, 33(3), 349–365. 10.1080/08856257.2017.1314111

[bibr35-13623613261426099] Ferguson L. (2021). Vulnerable children’s right to education, school exclusion, and pandemic law-making. Emotional and Behavioural Difficulties, 26(1), 101–115. 10.1080/13632752.2021.1913351

[bibr36-13623613261426099] García-Cedillo I. Romero-Contreras S. Ramos-Abadie L. (2015). Where do Mexico and Chile stand on inclusive education? International Journal of Special Education, 30(2), 145–156.

[bibr37-13623613261426099] Goodall C. (2018a). ‘I felt closed in and like I couldn’t breathe’: A qualitative study exploring the mainstream educational experiences of autistic young people. Autism & Developmental Language Impairments, 3, 2396941518804407. 10.1177/2396941518804407

[bibr38-13623613261426099] Goodall C. (2018b). Mainstream is not for all: The educational experiences of autistic young people. Disability & Society, 33(10), 1661–1665. 10.1080/09687599.2018.1529258

[bibr39-13623613261426099] Goodall C. (2019). ‘There is more flexibility to meet my needs’: Educational experiences of autistic young people in mainstream and alternative education provision. Support for Learning, 34(1), 4–33. 10.1111/1467-9604.12236

[bibr40-13623613261426099] Goodall C. MacKenzie A. (2019). What about my voice? Autistic young girls’ experiences of mainstream school. European Journal of Special Needs Education, 34(4), 499–513. 10.1080/08856257.2018.1553138

[bibr41-13623613261426099] Goodenow C. (1993). The psychological sense of school membership among adolescents: Scale development and educational correlates. Psychology in the Schools, 30(1), 79–90. 10.1002/1520-6807(199301)30:1<79::aid-pits2310300113>3.0.co;2-x

[bibr42-13623613261426099] Goodman R. (1997). The Strengths and Difficulties Question-naire: A research note. Journal of Child Psychology and Psychiatry, 38(5), 581–586. 10.1111/j.1469-7610.1997.tb01545.x 9255702

[bibr43-13623613261426099] Greenlee J. L. Mosley A. S. Shui A. M. Veenstra-VanderWeele J. Gotham K. O. (2016). Medical and behavioral correlates of depression history in children and adolescents with autism spectrum disorder. Pediatrics, 137(Suppl. 2), S105–S114. 10.1542/peds.2015-2851I PMC4915738 26908466

[bibr44-13623613261426099] Halsall J. Clarke C. Crane L. (2021). ‘Camouflaging’ by adolescent autistic girls who attend both mainstream and specialist resource classes: Perspectives of girls, their mothers and their educators. Autism, 25(7), 2074–2086. 10.1177/13623613211012819 33966484 PMC8419293

[bibr45-13623613261426099] Hamburger M. E. Basile K.C. Vivolo A. M. (2011). Measuring bullying victimization, perpetration, and bystander experiences; a compendium of assessment tools. Atlanta, GA: Centers for Disease Control and Prevention, National Center for Injury Prevention and Control. 10.1037/e580662011-001

[bibr46-13623613261426099] Hamilton L. G. Cook A. (2026). Supporting neurodivergent pupils in mainstream schools: A mixed-methods survey of school staff in the United Kingdom. Neurodiversity, 4. 10.1177/27546330261419277

[bibr47-13623613261426099] Hebron J. Bond C. (2017). Developing mainstream resource provision for pupils with autism spectrum disorder: Parent and pupil perceptions. European Journal of Special Needs Education, 32(4), 556–571. https://doi.org/j10.1080/08856257.2017.1297569

[bibr48-13623613261426099] Hebron J. Humphrey N. (2014). Mental health difficulties among young people on the autistic spectrum in mainstream secondary schools: A comparative study. Journal of Research in Special Educational Needs, 14(1), 22–32. 10.1111/j.1471-3802.2012.01246.x

[bibr49-13623613261426099] Hebron J. Humphrey N. Oldfield J. (2015). Vulnerability to bullying of children with autism spectrum conditions in mainstream education: A multi-informant qualitative exploration. Journal of Research in Special Educational Needs, 15(3), 185–193. 10.1111/1471-3802.12108

[bibr50-13623613261426099] Hill L. (2014). Some of it I haven’t told anybody else’: Using photo elicitation to explore the experiences of secondary school education from the perspective of young people with a diagnosis of Autistic Spectrum Disorder. Educational & Child Psychology, 31(1), 79–89. 10.53841/bpsecp.2014.31.1.79

[bibr51-13623613261426099] Horan M. Merrigan C. (2019). Teachers’ perceptions of the effect of professional development on their efficacy to teach pupils with ASD in special classes. REACH: Journal of Inclusive Education in Ireland, 32(1), 34–49.

[bibr52-13623613261426099] Horgan F. Kenny N. Flynn P. (2022). A systematic review of the experiences of autistic young people enrolled in mainstream second-level (post-primary) schools. Autism, 27(2), 526–538. 10.1177/13623613221105089 35757990

[bibr53-13623613261426099] Huebner E. S. (1997). Life satisfaction and happiness. In Bear G. Minke K. Thomas A. (Eds.), Children’s needs II: Development, problems, and alternatives (pp. 271–278), Silver Springs.

[bibr54-13623613261426099] Hull L. Petrides K. V. Mandy W. (2020). The female autism phenotype and camouflaging: A narrative review. Review Journal of Autism and Developmental Disorders, 7, 306–317. 10.1007/s40489-020-00197-9

[bibr55-13623613261426099] Humphrey N. Lewis S. (2008). Make me normal’ The views and experiences of pupils on the autistic spectrum in mainstream secondary schools. Autism, 12(1), 23–46. 10.1177/1362361307085267 18178595

[bibr56-13623613261426099] Humphrey N. Symes W. (2010). Perceptions of social support and experience of bullying among pupils with autistic spectrum disorders in mainstream secondary schools. European Journal of Special Needs Education, 25(1), 77–91. 10.1080/08856250903450855

[bibr57-13623613261426099] Ito H. Chang-Leung C. Poudyal H. (2023). Inclusion of students with developmental disabilities in Japan: Barriers and promising practices in primary and secondary education. Asia Pacific Education Review, 24(3), 415–431. 10.1007/s12564-022-09763-8

[bibr58-13623613261426099] Jennings P. A. Greenberg M. T. (2009). The prosocial classroom: Teacher social and emotional competence in relation to student and classroom outcomes. Review of Educational Research, 79(1), 491–525. 10.3102/0034654308325693

[bibr59-13623613261426099] Kasari C. Locke J. Gulsrud A. Rotheram-Fuller E. (2011). Social networks and friendships at school: Comparing children with and without ASD. Journal of Autism and Developmental Disorders, 41, 533–544. 10.1007/s10803-010-1076-x 20676748 PMC3076578

[bibr60-13623613261426099] Keane E. Aldridge F. J. Costley D. Clark T. (2012). Students with autism in regular classes: A long-term follow-up study of a satellite class transition model. International Journal of Inclusive Education, 16, 1001–1017.

[bibr61-13623613261426099] Keen D. Webster A. Ridley G. (2016). How well are children with autism spectrum disorder doing academically at school? An overview of the literature. Autism, 20(3), 276–294. 10.1177/1362361315580962 25948598

[bibr62-13623613261426099] Keith J. M. Jamieson J. P. Bennetto L. (2019). The importance of adolescent self-report in autism spectrum disorder: Integration of questionnaire and autonomic measures. Journal of Abnormal Child Psychology, 47, 741–754. 10.1007/s10802-018-0455-1 30073571 PMC6359986

[bibr63-13623613261426099] Keyes C. L. (2007). Promoting and protecting mental health as flourishing: A complementary strategy for improving national mental health. American Psychologist, 62(2), 95. 10.1037/0003-066X.62.2.95 17324035

[bibr64-13623613261426099] Kim J. A. Szatmari P. Bryson S. E. Streiner D. L. Wilson F. J. (2000). The prevalence of anxiety and mood problems among children with autism and Asperger syndrome. Autism, 4(2), 117–132.ur. 10.1177/1362361300004002002

[bibr65-13623613261426099] Krieger B. Piškur B. Schulze C. Jakobs U. Beurskens A. Moser A. (2018). Supporting and hindering environments for participation of adolescents diagnosed with autism spectrum disorder: A scoping review. PLOS ONE, 13(8), Article e0202071. 10.1371/journal.pone.0202071 PMC6114703 30157207

[bibr66-13623613261426099] Kurth J. A. Mastergeorge A. M. (2010). Academic and cognitive profiles of students with autism: Implications for classroom practice and placement. International Journal of Special Education, 25(2), 8–14.

[bibr67-13623613261426099] Lai M.-C. Kassee C. Besney R. Bonato S. Hull L. Mandy W. . . .Ameis S. H. (2019). Prevalence of co-occurring mental health diagnoses in the autism population: A systematic review and meta-analysis. The Lancet Psychiatry, 6(10), 819–829. 10.1016/S2215-0366(19)30289-5 31447415

[bibr68-13623613261426099] Lindsay G. Ricketts J. Peacey L. V. Dockerell J. E. Charman T. (2016). Meeting the educational and social needs of children with language impairment or autism spectrum disorder: The parent’s perspective. International Journal of Language and Communication Disorders, 51(5), 495–507. 10.1111/1460-6984.12226 26952185

[bibr69-13623613261426099] Lindsay S. Proulx M. Thomson N. Scott H. (2013). Educators’ challenges of including children with autism spectrum disorder in mainstream classrooms. International Journal of Disability, Development and Education, 60(4), 347–362. 10.1080/1034912X.2013.846470

[bibr70-13623613261426099] Lippman L. H. Moore K. A. Guzman L. Ryberg R. McIntosh H. Ramos M. F. . . .Kuhfeld M. (2014). Flourishing children: Defining and testing indicators of positive development. Springer. 10.1007/978-94-017-8607-2

[bibr71-13623613261426099] Liu W. C. Wang C. K. J. (2005). Academic self-concept: A cross-sectional study of grade and gender differences in a Singapore secondary school. Asia Pacific Education Review, 6(1), 20–27. 10.1007/bf03024964

[bibr72-13623613261426099] Locke J. Ishijima E. H. Kasari C. London N. (2010). Loneliness, friendship quality and the social networks of adolescents with high-functioning autism in an inclusive school setting. Journal of Research in Special Educational Needs, 10(2), 74–81. 10.1111/j.1471-3802.2010.01148.x

[bibr73-13623613261426099] Lyubomirsky S. Lepper H. S. (1999). A measure of subjective happiness: Preliminary reliability and construct validation. Social Indicators Research, 46(2), 137–155. 10.1023/a:1006824100041

[bibr74-13623613261426099] Maiano C. Normand C. L. Salvas M. C. Moullec G. Aime A. (2016). Prevalence of school bullying among youth with autism spectrum disorders: A systematic review and meta-analysis. Autism Research, 9(6), 601–615. 10.1002/aur.1568 26451871

[bibr75-13623613261426099] Malecki C. K. Demaray M. K. Elliott S. N. Nolten P. W. (2000). Child and adolescent social support scale. Psychology in the Schools. 10.1037/t57891-000

[bibr76-13623613261426099] Mandy W. Chilvers R. Chowdhury U. Salter G. Seigal A. Skuse D. (2012). Sex differences in autism spectrum disorder: Evidence from a large sample of children and adolescents. Journal of Autism and Developmental Disorders, 42, 1304–1313. 10.1007/s10803-011-1356-0 21947663

[bibr77-13623613261426099] Mayes S. D. Gorman A. A. Hillwig-Garcia J. Syed E. (2013). Suicide ideation and attempts in children with autism. Research in Autism Spectrum Disorders, 7(1), 109–119. 10.1016/j.rasd.2012.07.009

[bibr78-13623613261426099] McAllister K. Sloan S. (2016). Designed by the pupils, for the pupils: An autism-friendly school. British Journal of Special Education, 43(4), 330–357. 10.1111/1467-8578.12160

[bibr79-13623613261426099] McGinnity Á. Meltzer H. Ford T. Goodman R . (2005). Mental health of children and young people in Great Britain, 2004 (Vol. 175; Green H. , Ed.). Palgrave Macmillan.

[bibr80-13623613261426099] Merikangas K. R. He J. P. Burstein M. Swanson S. A. Avenevoli S. Cui L. . . .Swendsen J. (2010). Lifetime prevalence of mental disorders in US adolescents: Results from the National Comorbidity Survey Replication-Adolescent Supplement (NCS-A). Journal of the American Academy of Child & Adolescent Psychiatry, 49(10), 980–989. 10.1016/j.jaac.2010.05.017 20855043 PMC2946114

[bibr81-13623613261426099] Mertens J. Zane E. R. Neumeyer K. Grossman R. (2017). How anxious do you think I am? Relationship between state and trait anxiety in children with and without ASD during social tasks. Journal of Autism and Developmental Disorders, 47, 3692–3703. 10.1007/s10803-016-2979-y 28074356 PMC5503798

[bibr82-13623613261426099] Moore C. (2016). School report 2016. The National Autistic Society.

[bibr83-13623613261426099] National Autistic Society. (2015). The National Autistic Society specialist resource centres.

[bibr84-13623613261426099] National Autistic Society. (2021, November 9). School report 2021. National Autistic Society. https://www.autism.org.uk/what-we-do/news/school-report-2021

[bibr85-13623613261426099] Neal S. Frederickson N. (2016). ASD transition to mainstream secondary: A positive experience? Educational Psychology in Practice, 32(4), 355–373. 10.1080/02667363.2016.1193478

[bibr86-13623613261426099] Newcomb A. F. Bagwell C. L. (1995). Children’s friendship relations: A meta-analytic review. Psychological Bulletin, 117(2), 306. 10.1037/0033-2909.117.2.306

[bibr87-13623613261426099] Norwich B. (2007). Dilemmas of difference, inclusion and disability: International perspectives and future directions. Routledge.

[bibr88-13623613261426099] Petrina N. Carter M. Stephenson J. (2014). The nature of friendship in children with autism spectrum disorders: A systematic review. Research in Autism Spectrum Disorders, 8(2), 111–126. 10.1016/j.rasd.2013.10.016

[bibr89-13623613261426099] Pfeffer F. T. (2015). Equality and quality in education. A comparative study of 19 countries. Social Science Research, 51, 350–368. 10.1016/j.ssresearch.2014.09.004 25769872 PMC4359749

[bibr90-13623613261426099] Poon K. K. Soon S. Wong M. E. Kaur S. Khaw J. Ng Z. Tan C. S. (2014). What is school like? Perspectives of Singaporean youth with high-functioning autism spectrum disorders. International Journal of Inclusive Education, 18(10), 1069–1081. 10.1080/13603116.2012.693401

[bibr91-13623613261426099] Preece D. Jordan R. (2010). Obtaining the views of children and young people with autism spectrum disorders about their experience of daily life and social care support. British Journal of Learning Disabilities, 38(1), 10–20. 10.1111/j.1468-3156.2009.00548.x

[bibr92-13623613261426099] Richter M. Flavier E. Popa-Roch M. Clément C. (2020). Perceptions on the primary-secondary school transition from French students with autism spectrum disorder and their parents. European Journal of Special Needs Education, 35(2), 171–187. 10.1080/08856257.2019.1643145

[bibr93-13623613261426099] Ridgway K. Macmillan C. Demmer D. H. Hooley M. Hedley D. Westrupp E. Stokes M. A. (2024). Subjective wellbeing of autistic adolescents and young adults: A cross sectional study. Autism Research, 17(6), 1175–1186. 10.1002/aur.3139 38682234

[bibr94-13623613261426099] Roberts J. M. Keane E. Clark T. R. (2008). Making inclusion work: Autism spectrum Australia’s satellite class project. Teaching Exceptional Children, 41, 22–27.

[bibr95-13623613261426099] Robertson K. Chamberlain B. Kasari C. (2003). General education teachers’ relationships with included students with autism. Journal of Autism and Developmental Disorders, 33, 123–130. 10.1023/A:1022979108096 12757351

[bibr96-13623613261426099] Roorda D. L. Koomen H. M. Spilt J. L. Oort F. J. (2011). The influence of affective teacher-student relationships on students’ school engagement and achievement: A meta-analytic approach. Review of Educational Research, 81(4), 493–529. 10.3102/0034654311421793

[bibr97-13623613261426099] Saggers B. (2015). Student perceptions: Improving the educational experiences of high school students on the autism spectrum. Improving Schools, 18(1), 35–45. 10.1177/1365480214566213

[bibr98-13623613261426099] Saggers B. Hwang Y. S. Mercer K. L. (2011). Your voice counts: Listening to the voice of high school students with autism spectrum disorder. Australasian Journal of Special Education, 35(2), 173–190. 10.1375/ajse.35.2.173

[bibr99-13623613261426099] Sakiz G. Pape S. J. Hoy A. W. (2012). Does perceived teacher affective support matter for middle school students in mathematics classrooms? Journal of School Psychology, 50(2), 235–255. 10.1016/j.jsp.2011.10.005 22386122

[bibr100-13623613261426099] Seligman M. (2011). Flourish: A new understanding of happiness, well-being and how to achieve them. Nicholas Brealey Publishing.

[bibr101-13623613261426099] Shechtman Z. Leichtentritt J. (2004). Affective teaching: A method to enhance classroom management. European Journal of Teacher Education, 27(3), 323–333. 10.1080/0261976042000290822

[bibr102-13623613261426099] Shochet I. M. Dadds M. R. Ham D. Montague R. (2006). School connectedness is an underemphasized parameter in adolescent mental health: Results of a community prediction study. Journal of Clinical Child and Adolescent Psychology, 35(2), 170–179. 10.1207/s15374424jccp3502_1 16597213

[bibr103-13623613261426099] Solomon M. Miller M. Taylor S. L. Hinshaw S. P. Carter C. S. (2012). Autism symptoms and internalizing psychopathology in girls and boys with autism spectrum disorders. Journal of Autism and Developmental Disorders, 42, 48–59. 10.1007/s10803-011-1215-z 21442362 PMC3244604

[bibr104-13623613261426099] Tierney S. Burns J. Kilbey E. (2016). Looking behind the mask: Social coping strategies of girls on the autistic spectrum. Research in Autism Spectrum Disorders, 23, 73–83. 10.1016/j.rasd.2015.11.013

[bibr105-13623613261426099] Tobias A. (2009). Supporting students with autistic spectrum disorder (ASD) at secondary school: A parent and student perspective. Educational Psychology in Practice, 25(2), 151–166. 10.1080/02667360902905239

[bibr106-13623613261426099] United Nations Educational, Scientific & Cultural Organisation. (1994). The Salamanca statement and framework for action on special needs education. https://unesdoc.unesco.org/ark:/48223/pf0000098427

[bibr107-13623613261426099] United Nations Educational, Scientific & Cultural Organisation. (2009). Policy guidelines on inclusion in education. http://unesdoc.unesco.org/images/0017/001778/177849e.pdf

[bibr108-13623613261426099] van Harmelen A. L. Blakemore S. J. Goodyer I. M. Kievit R. A. (2021). The interplay between adolescent friendship quality and resilient functioning following childhood and adolescent adversity. Adversity and Resilience Science, 2(1), 37–50. 10.1007/s42844-020-00027-1 37915317 PMC7615274

[bibr109-13623613261426099] Van Steensel F. J. Bögels S. M. Perrin S . (2011). Anxiety disorders in children and adolescents with autistic spectrum disorders: A meta-analysis. Clinical Child and Family Psychology Review, 14, 302–317. 10.1007/s10567-011-0097-0 21735077 PMC3162631

[bibr110-13623613261426099] Warnock M. (2005, June 29). Architect of special needs inclusion calls for policy review. The Guardian. https://www.theguardian.com/education/2005/jun/29/schools.uk1

[bibr111-13623613261426099] Wentzel K. R. Battle A. Russell S. L. Looney L. B. (2010). Social supports from teachers and peers as predictors of academic and social motivation. Contemporary Educational Psychology, 35(3), 193–202. 10.1016/j.cedpsych.2010.03.002

