# Supplementary material for: The impact of specialist resource centres on autistic pupils’ experience of mainstream school
Source: Autism. 2026 Mar 13;30(4):1088–107. doi: 10.1177/13623613261426099 (PMC13005901; doi:10.1177/13623613261426099)
Supplement: sj-pdf-1-aut-10.1177_13623613261426099 – Supplemental material for The impact of specialist resource centres on autistic pupils’ experience of mainstream school [file sj-pdf-1-aut-10.1177_13623613261426099.pdf]

SUPPLEMENTARY MATERIAL

Table 9

Correlations Among Variables Associated with Internalising Symptoms (SDQ)

Variable	1	2	3	4	5	6	7	8
1. T4 Internalising Symptoms	—							
2. T1 Internalising Symptoms	.642***	—						
3. Gender	.419***	.259**	—					
4. Age at baseline	−.070	−.183*	−.107	—				
5. Teacher Support (T4)	−.180*	−.185*	−.136	−.092	—			
6. Peer Support (T4)	−.476***	−.175	−.085	−.035	.498***	—		
7. M-SRC dummy (vs. SRC)	−.188*	−.120	−.053	−.118	−.147	.079	—	
8. N-SRC dummy (vs. SRC)	.121	.016	.199*	.003	−.015	−.125	−.378***	—

Note. Values are Pearson correlations (one-tailed). *$p < .05$, **$p < .01$, ***$p < .001$.

Table 10

Hierarchical Regression Results for T4 Internalising Symptoms (SDQ)

Predictor	*B*	*SE B*	*β*	*t*	*p*	*R^2 (Adj. R^2)*
Step 1						0.412 (0.405)
T1 internalising symptoms (SDQ)	0.680	0.091	0.642	7.486	<.001	
Step 2 (add demographics)						0.485 (0.465)
T1 internalising symptoms (SDQ)	0.617	0.090	0.582	6.829	<.001	
Gender	2.605	0.798	0.275	3.264	.002	
Age at baseline	0.029	0.037	0.066	0.791	.431	
Step 3 (add perceived support)						0.636 (0.612)
T1 internalising symptoms (SDQ)	0.570	0.079	0.537	7.245	<.001	
Gender	2.587	0.683	0.273	3.785	<.001	
Age at baseline	0.026	0.032	0.059	0.821	.414	
Teacher support (T4)	0.076	0.033	0.185	2.279	.025	
Peer support (T4)	-0.165	0.029	-0.449	-5.595	<.001	
Step 4 (add placement)						0.638 (0.603)
T1 internalising symptoms (SDQ)	0.561	0.081	0.529	6.928	<.001	
Gender	2.589	0.706	0.274	3.667	<.001	
Age at baseline	0.022	0.032	0.050	0.686	.495	
Teacher support (T4)	0.071	0.035	0.172	2.024	.047	
Peer support (T4)	-0.162	0.030	-0.442	-5.355	<.001	
M-SRC dummy (vs. SRC)	-0.427	0.686	-0.050	-0.623	.535	
N-SRC dummy (vs. SRC)	-0.150	0.842	-0.014	-0.178	.859	

Note. *Dependent variable = T4 internalising symptoms. Step 1: $R^2 = .412$. Step 2: $\Delta R^2 = .073$, $p = .006$. Step 3: $\Delta R^2 = .151$, $p < .001$. Step 4: $\Delta R^2 = .002$, ns.*

Table 11

Correlations Among Variables Associated with Peer Problems (SDQ)

Variable	1	2	3	4	5	6	7	8
1. Time 4 Peer Problems	—	—	—	—	—	—	—	—
2. Time 1 Peer Problems	.504***	—	—	—	—	—	—	—
3. Gender	.164	.102	—	—	—	—	—	—
4. Age at baseline	.009	-.168	-.107	—	—	—	—	—
5. Teacher Support (T4)	-.174*	-.114	-.136	-.092	—	—	—	—
6. Peer Support (T4)	-.626***	-.178	-.085	-.035	.498***	—	—	—
7. M-SRC dummy (vs. SRC)	-.150	-.195*	-.053	-.118	-.147	.079	—	—
8. N-SRC dummy (vs. SRC)	.075	-.067	.199*	.003	-.015	-.125	-.378***	—

*Note. Correlations are Pearson's r (one-tailed). * $p < .05$. ** $p < .01$. *** $p < .001$.*

Table 12

Hierarchical Regression Results for T4 Peer Problems (SDQ)

Predictor	*B*	*SE B*	*β*	*t*	*p*	*R² (Adj. R²)*
**Step 1**						.254 (.244)
Time 1 peer problems	.556	.107	.504	5.216	<.001	
**Step 2 (add demographics)**						.278 (.250)
Time 1 peer problems	.562	.108	.509	5.198	<.001	
Gender	.631	.497	.123	1.270	.208	
Age at baseline	.026	.023	.108	1.101	.274	
**Step 3 (add perceived support)**						.596 (.570)
Time 1 peer problems	.461	.083	.417	5.529	<.001	
Gender	.539	.380	.105	1.420	.160	
Age at baseline	.021	.018	.088	1.179	.242	
Teacher support (T4)	.049	.019	.219	2.577	.012	
**Peer support (T4)**	-.129	.017	-.649	-7.643	<.001	
Step 4 (add placement)						.598 (.560)
Time 1 peer problems	.474	.088	.429	5.362	<.001	
Gender	.534	.393	.104	1.358	.178	
Age at baseline	.023	.018	.096	1.241	.218	
Teacher support (T4)	.051	.020	.229	2.582	.012	
Peer support (T4)	-.130	.017	-.653	-7.476	<.001	
M-SRC dummy (vs. SRC)	.200	.400	.043	.500	.618	
N-SRC dummy (vs. SRC)	.120	.487	.021	.248	.805	

Note. Dependent variable = Time 4 peer problems. β = standardized coefficient. Step 1: R^2 = .254. Step 2: ΔR^2 = .024, ns. Step 3: ΔR^2 = .319, p < .001. Step 4: ΔR^2 = .001, ns.

Table 13

Correlations Among Variables Associated with Emotional Problems (SDQ)

Variable	1	2	3	4	5	6	7	8
1. T4 Emotional Problems	—							
2. T1 Emotional Problems	.678***	—						
3. Gender	.513***	.321**	—					
4. Age at baseline	−.117	−.149	−.107	—				
5. Teacher Support (T4)	−.134	−.196*	−.136	−.092	—			
6. Peer Support (T4)	−.213*	−.130	−.085	−.035	.498***	—		
7. M-SRC dummy (vs. SRC)	−.166*	−.029	−.053	−.118	−.147	.079	—	
8. N-SRC dummy (vs. SRC)	.124	.079	.199*	.003	−.015	−.125	−.378***	—

*Note. *p* < .05. **p** < .01. ***p*** < .001 (one-tailed).*

Table 14

Hierarchical Regression Results for T4 Emotional Problems (SDQ)

Predictor	B	SE B	*β*	t	p	R² (Adj. R²)
**Step 1**						0.460 (0.453)
T1 Emotional Problems	0.723	0.088	0.678	8.256	< .001	
**Step 2 (add demographics)**						0.557 (0.540)
T1 Emotional Problems	0.611	0.085	0.573	7.148	< .001	
Gender	2.007	0.487	0.329	4.124	< .001	
Age at baseline	0.001	0.022	0.004	0.049	.961	
**Step 3 (add perceived support)**						0.577 (0.550)
T1 Emotional Problems	0.610	0.086	0.573	7.102	< .001	
Gender	2.014	0.483	0.330	4.166	< .001	
Age at baseline	0.002	0.022	0.008	0.102	.919	
Teacher Support (T4)	0.028	0.023	0.105	1.200	.234	
Peer Support (T4)	−0.039	0.020	−0.163	−1.893	.062	
**Step 4 (add placement)**						0.591 (0.552)
T1 Emotional Problems	0.604	0.086	0.566	7.033	< .001	
Gender	2.019	0.491	0.331	4.115	< .001	
Age at baseline	−0.003	0.022	−0.011	−0.137	.891	
Teacher Support (T4)	0.019	0.024	0.073	0.814	.418	
Peer Support (T4)	−0.034	0.021	−0.145	−1.656	.102	
M-SRC dummy (vs. SRC)	−0.726	0.462	−0.131	−1.571	.120	
N-SRC dummy (vs. SRC)	−0.368	0.573	−0.053	−0.642	.523	

Note. Dependent variable = T4 Emotional Problems. Step 1: $R^2 = .460$. Step 2: $\Delta R^2 = .097$, $p < .001$. Step 3: $\Delta R^2 = .021$, ns. Step 4: $\Delta R^2 = .014$, ns.

Table 15

Correlations Among Variables Associated with T3 Academic Self-Concept

Variable	1	2	3	4	5	6	7	8
1. T3 Academic Self-Concept	—							
2. T1 Academic Self-Concept	.496***	—						
3. Gender	-.279**	-.123	—					
4. Age at baseline	.146	.061	-.107	—				
5. Teacher Support (T3)	.343***	.300*	-.136	-.092	—			
6. Peer Support (T3)	.205*	.181	-.085	-.035	.498***	—		
7. M-SRC dummy (vs. SRC)	.063	.123	-.053	-.118	-.147	.079	—	
8. N-SRC dummy (vs. SRC)	-.029	.181	.199*	.003	-.015	-.125	-.378***	—

Note. Values are Pearson correlations (one-tailed). *p < .05. **p < .01. ***p < .001.

Table 16

Hierarchical Regression Results for T3 Academic Self-Concept

Predictor	*B*	*SE B*	*β*	*t*	*p*	*R^2 (Adj. R^2)*
**Step 1**						0.246 (0.233)
T1 academic self-concept	.215	.049	.496	4.353	< .001	
**Step 2 (add demographics)**						0.303 (0.266)
T1 academic self-concept	.201	.049	.464	4.126	< .001	
Gender	-1.477	.787	-.212	-1.878	.066	
Age at baseline	.031	.037	.095	.842	.403	
**Step 3 (add perceived support)**						0.342 (0.281)
T1 academic self-concept	.175	.051	.403	3.452	.001	
Gender	-1.312	.784	-.188	-1.673	.100	
Age at baseline	.039	.036	.120	1.068	.290	
Teacher support (T3)	.060	.040	.197	1.486	.143	
Peer support (T3)	.006	.034	.022	.171	.865	
**Step 4 (add placement)**						0.346 (0.258)
T1 academic self-concept	.178	.055	.410	3.230	.002	
Gender	-1.215	.817	-.174	-1.488	.143	
Age at baseline	.041	.038	.125	1.077	.287	
Teacher support (T3)	.063	.043	.207	1.471	.147	
Peer support (T3)	.002	.036	.008	.059	.953	
M-SRC dummy (vs. SRC)	.170	.830	.027	.205	.838	
N-SRC dummy (vs. SRC)	-.432	1.039	-.054	-.416	.679	

Note. Dependent variable = T3 academic self-concept. Step 1: $R^2 = .246$. Step 2: $\Delta R^2 = .057$, ns. Step 3: $\Delta R^2 = .039$, ns. Step 4: $\Delta R^2 = .004$, ns.

Table 17

Correlations Among Variables Associated with T3 Subjective Happiness

Variable	1	2	3	4	5	6	7	8
1. T3 subjective happiness	—							
2. T1 subjective happiness	.469***	—						
3. Gender	−.240**	−.199	—					
4. Age at baseline	−.021	−.037	−.107	—				
5. Teacher support (T4)	.403***	.268*	−.136	−.092	—			
6. Peer support (T4)	.470***	.042	−.085	−.035	.498***	—		
7. M-SRC dummy (vs. SRC)	.039	.028	−.053	−.118	−.147	.079	—	
8. N-SRC dummy (vs. SRC)	−.074	.022	.199*	.003	−.015	−.125	−.378***	—

Note. Values are Pearson correlations (one-tailed). *** p < .001, ** p < .01, * p < .05.

Table 18

Hierarchical Regression Results for T3 Subjective Happiness

Predictor	*B*	*SE B*	*β*	*t*	*p*	*R² (Adj. R²)*
**Step 1**						.220 (.207)
T1 subjective happiness	0.529	0.132	0.469	4.012	<.001	
**Step 2 (add demographics)**						.243 (.202)
T1 subjective happiness	0.493	0.135	0.438	3.648	<.001	
Gender	-1.881	1.465	-0.155	-1.284	.205	
Age at baseline	-0.012	0.067	-0.021	-0.181	.857	
**Step 3 (add perceived support)**						.440 (.388)
T1 subjective happiness	0.461	0.123	0.409	3.753	<.001	
Gender	-1.374	1.290	-0.113	-1.065	.292	
Age at baseline	0.002	0.059	0.003	0.029	.977	
Teacher support (T4)	0.041	0.066	0.077	0.618	.539	
Peer support (T4)	0.191	0.056	0.405	3.395	.001	
**Step 4 (add placement)**						.441 (.364)
T1 subjective happiness	0.462	0.126	0.410	3.664	<.001	
Gender	-1.353	1.344	-0.111	-1.007	.319	
Age at baseline	0.002	0.061	0.003	0.028	.978	
Teacher support (T4)	0.041	0.069	0.077	0.588	.559	
Peer support (T4)	0.191	0.059	0.404	3.258	.002	
M-SRC dummy (vs. SRC)	-0.024	1.302	-0.002	-0.018	.986	
N-SRC dummy (vs. SRC)	-0.127	1.613	-0.009	-0.079	.937	

Note. Dependent variable = T3 subjective happiness. Step 1: $R^2 = .220$. Step 2: $\Delta R^2 = .023$, ns. Step 3: $\Delta R^2 = .198$, $p < .001$. Step 4: $\Delta R^2 < .001$, ns.

Table 19

Correlations Among Variables Used in Analysis Associated with Life Satisfaction

Variable	1	2	3	4	5	6	7	8
1. T3 Life Satisfaction	—							
2. T1 Life Satisfaction	.459***	—						
3. Gender	−.300**	−.204*	—					
4. Age at baseline	.016	.096	−.107	—				
5. Teacher support (T4)	.501***	.376**	−.136	−.092	—			
6. Peer support (T4)	.445***	.346**	−.085	−.035	.498***	—		
7. M-SRC dummy (vs. SRC)	−.095	−.006	−.053	−.118	−.147	.079	—	
8. N-SRC dummy (vs. SRC)	.015	.075	.199*	.003	−.015	−.125	−.378***	—

Note. One-tailed Pearson correlations. *$p < .05$. **$p < .01$. ***$p < .001$.

Table 20

Hierarchical Regression Results for T3 Life Satisfaction

Predictor	*B*	*SE B*	*β*	*t*	*p*	*R² (Adj. R²)*
Step 1						0.211 (0.197)
T1 Life Satisfaction	0.466	0.118	0.459	3.939	< .001	
Step 2 (add demographics)						0.258 (0.218)
T1 Life Satisfaction	0.426	0.120	0.419	3.554	< .001	
Gender	-2.116	1.139	-0.219	−1.857	.069	
Age at baseline	-0.021	0.052	-0.048	−0.410	.684	
Step 3 (add perceived support)						0.402 (0.347)
T1 Life Satisfaction	0.246	0.120	0.242	2.048	.045	
Gender	-1.872	1.044	-0.194	−1.792	.079	

Age at baseline	0.002	0.048	0.005	0.048	.962	
Teacher support (T4)	0.118	0.053	0.283	2.236	.030	
Peer support (T4)	0.076	0.046	0.204	1.645	.106	
Step 4 (add placement)						0.410 (0.331)
T1 Life Satisfaction	0.240	0.124	0.237	1.944	.057	
Gender	-2.028	1.084	-0.210	−1.870	.067	
Age at baseline	-0.002	0.050	-0.005	−0.048	.962	
Teacher support (T4)	0.110	0.055	0.262	1.984	.053	
Peer support (T4)	0.084	0.048	0.226	1.763	.084	
M-SRC dummy (vs. SRC)	-0.593	1.050	-0.067	−0.565	.575	
N-SRC dummy (vs. SRC)	0.508	1.318	0.046	0.385	.701	

Note. Dependent variable = T3 Life Satisfaction. *B* = unstandardized coefficient; *β* = standardized coefficient. Step 1: $R^2 = .211$. Step 2: $\Delta R^2 = .047$, ns. Step 3: $\Delta R^2 = .145$, p = .003. Step 4: $\Delta R^2 = .008$, ns.

Table 21

Correlations Among Variables Used in Analyses Associated with Flourishing

Variable	1	2	3	4	5	6	7	8
1. Time 3 Flourishing	—	—	—	—	—	—	—	—
2. Time 1 Flourishing	.399**	—	—	—	—	—	—	—
3. Gender	-.161	-.225*	—	—	—	—	—	—
4. Age at baseline	.088	.063	-.107	—	—	—	—	—
5. Teacher Support (T4)	.586***	.298*	-.136	-.092	—	—	—	—
6. Peer Support (T4)	.542***	.193	-.085	-.035	.498***	—	—	—
7. M-SRC dummy (vs. SRC)	-.035	.107	-.053	-.118	-.147	.079	—	—
8. N-SRC dummy (vs. SRC)	.060	.213*	.199*	.003	-.015	-.125	-.378***	—

Note. *r* values are Pearson correlations (one-tailed). * p < .05, ** p < .01, *** p < .001.

Table 22

Hierarchical Regression Results for Time 3 Flourishing

Predictor	*B*	*SE B*	*β*	*t*	*p*	*R^2 (Adj. R^2)*
**Step 1**						0.159 (0.145)
Time 1 flourishing	.449	.135	.399	3.316	.002	
Step 2 (add demographics)						0.168 (0.123)
**Time 1 flourishing**	.427	.141	.380	3.034	.004	
Gender	-.961	1.736	-.070	-.554	.582	
Age at baseline	.037	.079	.057	.464	.644	
**Step 3 (add perceived support)**						0.490 (0.442)
Time 1 flourishing	.242	.117	.215	2.060	.044	
Gender	-.319	1.390	-.023	-.229	.820	
Age at baseline	.076	.064	.117	1.189	.240	
Teacher support (T4)	.222	.070	.371	3.185	.002	
Peer support (T4)	.171	.060	.318	2.834	.006	
**Step 4 (add placement)**						0.495 (0.426)
Time 1 flourishing	.211	.130	.188	1.627	.110	
Gender	-.593	1.460	-.043	-.406	.686	
Age at baseline	.077	.066	.119	1.175	.245	
Teacher support (T4)	.225	.074	.375	3.043	.004	
Peer support (T4)	.176	.062	.329	2.831	.007	
M-SRC dummy (vs. SRC)	.215	1.448	.017	.149	.882	
N-SRC dummy (vs. SRC)	1.293	1.853	.082	.697	.489	

Note. Dependent variable = Time 3 flourishing. Step 1: $R^2 = .159$, *p* = .002; Step 2: $\Delta R^2 = .009$, ns; Step 3: $\Delta R^2 = .322$, *p* < .001; Step 4: $\Delta R^2 = .005$, ns.

Table 23

Correlations Among Variables Used in Analyses Associated with Sense of School Belonging

Variable	1	2	3	4	5	6	7	8
1. Time 4 Sense of Belonging	—							
2. Time 2 Sense of Belonging	.605***	—						

3. Gender	−.157	−.205*	—					
4. Age at baseline	−.086	−.101	−.107	—				
5. Teacher Support (T4)	.744***	.524***	−.136	−.092	—			
6. Peer Support (T4)	.624***	.406***	−.085	−.035	.498***	—		
7. M-SRC dummy (vs. SRC)	−.014	.027	−.053	−.118	−.147	.079	—	
8. N-SRC dummy (vs. SRC)	−.299**	−.016	.199*	.003	−.015	−.125	−.378***	—

*Note. Values are Pearson correlations (one-tailed). * p < .05. ** p < .01. *** p < .001.*

Table 24

Hierarchical Regression Results for Time 4 Sense of School Belonging

Predictor	*B*	*SE B*	*β*	*t*	*p*	*R² (Adj. R²)*
**Step 1**						0.366 (0.357)
Time 2 sense of belonging	0.627	0.099	0.605	6.316	<.001	
**Step 2 (add demographics)**						0.368 (0.340)
Time 2 sense of belonging	0.615	0.104	0.594	5.944	<.001	
Gender	-1.233	3.184	-0.039	-0.387	.700	
Age at baseline	-0.045	0.146	-0.031	-0.31	.757	
**Step 3 (add perceived support)**						0.680 (0.655)
Time 2 sense of belonging	0.242	0.088	0.234	2.735	.008	
Gender	-0.686	2.304	-0.022	-0.298	.767	
Age at baseline	-0.017	0.106	-0.012	-0.162	.872	
Teacher support (T4)	0.653	0.123	0.473	5.327	<.001	
Peer support (T4)	0.36	0.102	0.292	3.531	<.001	
**Step 4 (add placement)**						0.751 (0.723)
Time 2 sense of belonging	0.268	0.08	0.259	3.361	.001	
Gender	1.076	2.111	0.034	0.51	.612	
Age at baseline	-0.02	0.096	-0.014	-0.213	.832	
Teacher support (T4)	0.644	0.113	0.466	5.675	<.001	
Peer support (T4)	0.32	0.093	0.259	3.437	.001	
M-SRC dummy (vs. SRC)	-2.441	2.048	-0.084	-1.192	.238	
N-SRC dummy (vs. SRC)	-10.679	2.539	-0.294	-4.206	<.001	

Note. *Dependent variable = Time 4 sense of school belonging. Step 1: $R^2 = .366$. Step 2: $\Delta R^2 = .002$, ns. Step 3: $\Delta R^2 = .311, p < .001$. Step 4: $\Delta R^2 = .071, p < .001$.*

Table 25

Correlations Among Variables Used in Analyses Associated with Friendship Satisfaction

Variable	1	2	3	4	5	6	7	8
1. Time 4 Friendship	—							
2. Time 2 Friendship	.332**	—						
3. Gender	-.197*	-.021	—					
4. Age at baseline	.061	.232**	-.107	—				
5. Teacher Support (T4)	.157	.034	-.136	-.092	—			
6. Peer Support (T4)	.505***	.082	-.085	-.035	.498***	—		
7. M-SRC dummy (vs. SRC)	.070	-.168*	-.053	-.118	-.147	.079	—	
8. N-SRC dummy (vs. SRC)	-.127	.115	.199*	.003	-.015	-.125	-.378***	—

Note. Values are Pearson correlations. * p < .05, ** p < .01, *** p < .001 (1-tailed).

Table 26

Hierarchical Regression Results for Time 4 Friendship

**Predictor**	*B*	*SE B*	*β*	*t*	*p*	*R² (Adj. R²)*

**Step 1**						.110 (.097)
Time 2 friendship	.267	.091	.332	2.942	.004**	
**Step 2 (add demographics)**						.147 (.110)
Time 2 friendship	.271	.093	.336	2.923	.005**	
Gender	-1.697	.986	-.194	-1.721	.090	
Age at baseline	-.016	.047	-.038	-.328	.744	
**Step 3 (add perceived support)**						.379 (.331)
Time 2 friendship	.236	.081	.294	2.932	.005**	
Gender	-1.462	.864	-.167	-1.692	.095	
Age at baseline	-.008	.041	-.020	-.197	.844	
Teacher support (T4)	-.056	.043	-.146	-1.292	.201	
Peer support (T4)	.183	.038	.539	4.804	<.001***	
**Step 4 (add placement)**						.383 (.316)
Time 2 friendship	.246	.083	.306	2.970	.004**	
Gender	-1.340	.893	-.153	-1.501	.138	
Age at baseline	-.007	.042	-.017	-.166	.869	
Teacher support (T4)	-.051	.045	-.134	-1.133	.261	
Peer support (T4)	.178	.039	.524	4.512	<.001***	
M-SRC dummy (vs. SRC)	.225	.881	.028	.255	.800	
N-SRC dummy (vs. SRC)	-.576	1.089	-.058	-.529	.599	

Note. Dependent variable = Time 4 friendship. Step 1: $R^2 = .110$; Step 2: $\Delta R^2 = 0.037$, $p = .232$; Step 3: $\Delta R^2 = 0.231$, $p < .001$; Step 4: $\Delta R^2 = 0.005$, $p = .779$.

Table 27

Correlations Among Variables Associated with Victimisation

Variable	1	2	3	4	5	6	7	8
1. Time 4 total victimisation	—							
2. Time 2 total victimisation	.497***	—						
3. Gender	−.048	.155	—					
4. Age at baseline	−.066	−.093	−.107	—				
5. Teacher support (T4)	−.127	−.275**	−.136	−.092	—			
6. Peer support (T4)	−.238*	−.365***	−.085	−.035	.498***	—		
7. M-SRC dummy (vs. SRC)	−.177	−.184*	−.053	−.118	−.147	.079	—	
8. N-SRC dummy (vs. SRC)	.135	.149	.199*	.003	−.015	−.125	−.378***	—

Note. Values are Pearson's *r* (one-tailed). *p* < .05, **p* < .01, ***p* < .001.

Table 28

Hierarchical Regression Results for Time 4 Victimisation

Predictor	*B*	*SE B*	*β*	*t*	*p*	*R^2 (Adj. R^2)*
**Step 1**						*0.247 (0.236)*
Time 2 victimisation	*.451*	*.094*	*.497*	*4.821*	*<.001*	
**Step 2 (add demographics)**						*0.264 (0.232)*
Time 2 victimisation	*.467*	*.095*	*.514*	*4.901*	*<.001*	
Gender	*−2.816*	*2.247*	*−.132*	*−1.253*	*.215*	
Age at baseline	*−.032*	*.104*	*−.032*	*−.310*	*.757*	
**Step 3 (add perceived support)**						*0.269 (0.214)*
Time 2 victimisation	*.446*	*.104*	*.491*	*4.283*	*<.001*	
Gender	*−2.813*	*2.287*	*−.131*	*−1.230*	*.223*	
Age at baseline	*−.035*	*.106*	*−.035*	*−.327*	*.745*	
Teacher support (T4)	*.027*	*.114*	*.029*	*.236*	*.814*	
Peer support (T4)	*−.071*	*.104*	*−.085*	*−.678*	*.500*	
**Step 4 (add placement)**						*0.280 (0.202)*
Time 2 victimisation	*.425*	*.107*	*.467*	*3.955*	*<.001*	
Gender	*−3.155*	*2.347*	*−.147*	*−1.345*	*.183*	

Age at baseline	*−.050*	*.109*	*−.050*	*−.460*	*.647*
Teacher support (T4)	*.000*	*.119*	*.000*	*−.003*	*.997*
Peer support (T4)	*−.056*	*.106*	*−.068*	*−.530*	*.598*
M-SRC dummy (vs. SRC)	*−1.525*	*2.333*	*−.078*	*−.653*	*.516*
N-SRC dummy (vs. SRC)	*1.386*	*2.846*	*.057*	*.487*	*.628*

Note. Dependent variable = Time 4 total victimisation. Step 1: *R*² = .247, *p* < .001. Step 2: Δ*R*² = .017, ns. Step 3: Δ*R*² = .005, ns. Step 4: Δ*R*² = .011, ns.

Table 29

Correlations Among Variables Associated with Bullying

Variable	1	2	3	4	5	6	7	8
1. Time 4 Total Bullying	—							
2. Time 2 Total Bullying	.515***	—						
3. Gender	-.111	-.036	—					
4. Age at baseline	-.006	.018	-.107	—				
5. Teacher support (T4)	-.038	-.206*	-.136	-.092	—			
6. Peer support (T4)	.004	-.174*	-.085	-.035	.498***	—		
7. M-SRC dummy (vs. SRC)	-.151	-.170*	-.053	-.118	-.147	.079	—	
8. N-SRC dummy (vs. SRC)	-.136	-.092	.199*	.003	-.015	-.125	-.378***	—

Note. * p < .05. ** p < .01. *** p < .001 (one-tailed).

Table 30

Hierarchical Regression Results for Time 4 Bullying

Predictor	*B*	*SE B*	*β*	*t*	*p*	*R² (Adj. R²)*
**Step 1**						0.265 (0.254)
Time 2 bullying	.479	.095	.515	5.021	<.001	
**Step 2 (add demographics)**						0.274 (0.242)
Time 2 bullying	.476	.096	.512	4.948	<.001	
Gender	-1.419	1.537	-.096	-.923	.359	
Age at baseline	-.018	.072	-.026	-.251	.802	
**Step 3 (add perceived support)**						0.282 (0.227)
Time 2 bullying	.493	.100	.529	4.936	<.001	
Gender	-1.265	1.572	-.086	-.805	.424	
Age at baseline	-.014	.073	-.021	-.198	.843	
Teacher support (T4)	.012	.079	.018	.150	.882	
Peer support (T4)	.045	.069	.079	.653	.516	
**Step 4 (add placement)**						0.297 (0.221)
Time 2 bullying	.458	.104	.493	4.395	<.001	
Gender	-1.131	1.610	-.077	-.703	.485	
Age at baseline	-.025	.074	-.036	-.339	.736	
Teacher support (T4)	-.008	.082	-.012	-.094	.925	
Peer support (T4)	.048	.071	.084	.678	.500	
M-SRC dummy (vs. SRC)	-1.700	1.632	-.126	-1.042	.301	
N-SRC dummy (vs. SRC)	-1.905	1.992	-.113	-.956	.343	

Note. Dependent variable = Time 4 bullying. Step 1: R^2 = .265. Step 2: ΔR^2 = .009, ns. Step 3: ΔR^2 = .008, ns. Step 4: ΔR^2 = .016, ns.

Codebook

Study Overview

This codebook describes the variables, scales, and derived measures used in the study examining psychological wellbeing, social inclusion, perceived support, educational progress, and attendance/exclusions among autistic and non-autistic pupils in mainstream secondary schools with and without specialist resource centres (SRCs) in South-East England. The codebook is provided to support transparency and reproducibility. Raw data are not publicly available due to ethical and confidentiality restrictions.

Dataset Structure

- **Unit of analysis:** Pupil-level for survey and attainment data; school-level aggregated data for attendance and exclusions
- **Design:** Mixed factorial (placement × time) with additional correlational/regression analyses
- **Format:** Wide format with repeated measures indicated by timepoint suffixes (T1–T4)
- **Timepoints:**
  - T1 = Beginning of Year 1 (baseline)
  - T2 = End of Year 1
  - T3 = End of Year 2
  - T4 = End of Year 3

Key Grouping Variables

Variable name	Label	Type	Coding	Notes
Neurotype	Autism status	Binary	1 = Autistic<br>2 = Non-autistic	Confirmed by SRC lead or SENCo
Placement	School placement type	Categorical	1 = SRC<br>2 = M-SRC<br>3 = N-SRC	Primary between-groups factor
Gender	Gender	Categorical	0 = Male<br>1 = Female<br>2 = Non-binary<br>3 = Undisclosed	Self-reported
Age_T1	Age at baseline	Continuous	Months	Mean baseline age reported
EHCP	EHCP status	Binary	1 = EHCP<br>0 = No EHCP	Used as covariate

<u>Psychological Wellbeing Measures</u>

*[Note. * indicates variables measured at multiple timepoints.]*

Internalising Symptoms (SDQ; Goodman, 1997)

- Variable names: Total_internalising*, Total_Peer_Problems*, Total_Emotional_Problems*
- Description: Emotional problems and peer problems subscales of the Strengths and Difficulties Questionnaire
- Items: 10 (5 emotional, 5 peer problems)
- Response scale: 0 = Not true; 1 = Somewhat true; 2 = Certainly true
- Scoring: Subscales summed (0–10); internalising total summed (0–20)
- Reverse-coded items: Two reverse code items in peer problems sub-scale
- Interpretation: Higher scores indicate more difficulties
- Reliability: α = .86

Subjective Happiness (Lyubomirsky & Lepper, 1999)

- Variable name: Total_SubHap*
- Scale: Subjective Happiness Scale (4 items)
- Response scale: 1–7 (item-specific anchors)
- Scoring: Sum (4–28)
- Reverse-coded items: One item
- Interpretation: Higher scores indicate greater happiness
- Reliability: α = .85

Life Satisfaction (Huebner, 1997)
- Variable name: Total_LifeSat*
- Scale: Brief Multidimensional Students' Life Satisfaction Scale (6 items)
- Response scale: 1 = Very dissatisfied to 5 = Very satisfied
- Scoring: Sum (6–30)
- Reverse-coded items: None
- Interpretation: Higher scores indicate greater life satisfaction
- Reliability: α = .83

Academic Self-Concept (Liu & Wang, 2005)
- Variable name: Total_AcSelfConcept*
- Scale: Academic Self-Concept Scale (10 items)
- Response scale: 1 = Strongly disagree to 5 = Strongly agree
- Scoring: Sum (10–50)
- Reverse-coded items: None
- Interpretation: Higher scores indicate more positive academic self-concept
- Reliability: α = .88

Flourishing (Diener et al., 2009)
- Variable name: Total_Flourishing*
- Scale: Flourishing Scale (8 items)
- Response scale: 1 = Strongly disagree to 5 = Strongly agree
- Scoring: Sum (8–40)
- Reverse-coded items: None
- Interpretation: Higher scores indicate greater psychological flourishing
- Reliability: α = .87

Social Inclusion and Belonging

Friendship Quality (Goodyer et al., 1989)
- Variable name: Total_Friendhip*
- Scale: Cambridge Hormones and Moods Project Questionnaire (5 items)
- Response scale: 1 = Very unhappy to 5 = Very happy
- Scoring: Sum (5–25)
- Reverse-coded items: None
- Interpretation: Higher scores indicate better friendship quality

Bullying and Victimisation (Orpinas & Horne, 2006)
- Variable names: Total_Aggression*, Total_Victim*
- Scale: Reduced Aggression and Victimisation Scales (12 items)
- Response scale: 0–6 (frequency-based)
- Scoring: Sum per subscale (0–36)
- Reverse-coded items: None
- Interpretation: Higher scores indicate more frequent bullying/victimisation
- Reliability: Victimisation α = .89; Bullying α = .87

School Belonging (Goodenow, 1993)
- Variable name: Total_Belonging*
- Scale: Psychological Sense of School Membership (18 items)
- Response scale: 1–5 Likert

- Scoring: Sum (18–90)
- Reverse-coded items: Items negatively worded in the original scale were reverse-coded prior to scoring
- Interpretation: Higher scores indicate stronger sense of belonging
- Reliability: α = .95

<u>Perceived Support</u>

Peer Support (Malecki et al., 1999)
- Variable name: Total_Peer_Support*
- Scale: Child and Adolescent Social Support Scale – Peer subscale (10 items)
- Response scale: 1 = Never to 6 = Always
- Scoring: Sum (10–60)
- Reverse-coded items: None
- Interpretation: Higher scores indicate greater perceived peer support
- Reliability: α = .93

Teacher Support (Malecki et al., 1999)
- Variable name: Total_Peer_Support*
- Scale: Child and Adolescent Social Support Scale – Teacher subscale (10 items)
- Response scale: 1 = Never to 6 = Always
- Scoring: Sum (10–60)
- Reverse-coded items: None
- Interpretation: Higher scores indicate greater perceived teacher support
- Reliability: α = .92

<u>Educational Outcomes</u>

Academic Attainment
- Variable name: Teacher_Assessment_Average*
- Source: Teacher assessments in Mathematics, English, and Science
- Response scale: 1 = Below expected; 2 = Expected; 3 = Above expected
- Scoring: Mean across subjects per pupil
- Availability: T1–T3 only; N = 83

<u>Attendance and Exclusions (School-Level Aggregated Data)</u>

Attendance
- Variable names: Attendance_all, Attendance_autism, Attendance_SRC
- Description: Annual percentage attendance
- Unit: School-by-timepoint data points
- Derivation: Averaged across available years
- Derived variable: attendance_gap = subgroup attendance − whole-school attendance (negative values indicate lower attendance than school average)

Exclusions
- Variable names: Exclusions_count, Exclusion_days
- Description: Number and proportion of fixed-term exclusions and days lost
- Level: School-level aggregated
- Interpretation: Descriptive only; not individual-level

Overview of Data/Measures Used in Analysis by Time Point

Measure	T1	T2	T3	T4
Demographics	✓			
SDQ	✓	✓	✓	
Peer Support	✓	✓	✓	
Teacher Support	✓	✓	✓	
Academic Self Concept	✓		✓	
Subjective Happiness	✓		✓	
Life Satisfaction	✓		✓	
Flourishing	✓		✓	
Sense of School Belonging		✓		✓
Friendship Satisfaction		✓		✓
Bullying & Victimisation		✓		✓
Teacher Assessments	✓	✓	✓	

Derived and Analytical Variables

- Change scores: Follow-up minus baseline values
- Residualised change: Baseline score entered at Step 1 of regression models
- Handling missing data: Mean imputation when ≤25% of items missing; otherwise pairwise deletion

Software

All analyses were conducted using IBM SPSS Statistics (Version 29).
